# *In Silico* Analyses on the Comparative Potential of Therapeutic Human Monoclonal Antibodies Against Newly Emerged SARS-CoV-2 Variants Bearing Mutant Spike Protein

**DOI:** 10.3389/fimmu.2021.782506

**Published:** 2022-01-10

**Authors:** Nabarun Chandra Das, Pritha Chakraborty, Jagadeesh Bayry, Suprabhat Mukherjee

**Affiliations:** ^1^ Integrative Biochemistry and Immunology Laboratory, Department of Animal Science, Kazi Nazrul University, Asansol, India; ^2^ Department of Biological Sciences and Engineering, Indian Institute of Technology Palakkad, Palakkad, India

**Keywords:** COVID-19, SARS-CoV-2, variants, spike protein, monoclonal antibody (mAb), *in silico* approach

## Abstract

Since the start of the pandemic, SARS-CoV-2 has already infected more than 250 million people globally, with more than five million fatal cases and huge socio-economic losses. In addition to corticosteroids, and antiviral drugs like remdesivir, various immunotherapies including monoclonal antibodies (mAbs) to S protein of SARS-CoV-2 have been investigated to treat COVID-19 patients. These mAbs were initially developed against the wild-type SARS-CoV-2; however, emergence of variant forms of SARS-CoV-2 having mutations in the spike protein in several countries including India raised serious questions on the potential use of these mAbs against SARS-CoV-2 variants. In this study, using an *in silico* approach, we have examined the binding abilities of eight mAbs against several SARS-CoV-2 variants of Alpha (B.1.1.7) and Delta (B.1.617.2) lineages. The structure of the Fab region of each mAb was designed *in silico* and subjected to molecular docking against each mutant protein. mAbs were subjected to two levels of selection based on their binding energy, stability, and conformational flexibility. Our data reveal that tixagevimab, regdanvimab, and cilgavimab can efficiently neutralize most of the SARS-CoV-2 Alpha strains while tixagevimab, bamlanivimab, and sotrovimab can form a stable complex with the Delta variants. Based on these data, we have designed, by *in silico*, a chimeric antibody by conjugating the CDRH3 of regdanivimab with a sotrovimab framework to combat the variants that could potentially escape from the mAb-mediated neutralization. Our finding suggests that though currently available mAbs could be used to treat COVID-19 caused by the variants of SARS-CoV-2, better results could be expected with the chimeric antibodies.

## Introduction

The coronavirus disease 2019 (COVID-19) pandemic caused by severe acute respiratory syndrome coronavirus 2 (SARS-CoV-2) has become the biggest threat of the century to mankind with huge mortality (more than 5.1 million), socio-economic loss, and psychological issues (1–3). The causative virus SARS-CoV-2 is a spherical-shaped RNA virus surrounded by a glycoprotein envelope consisting of a crown-like spike protein alongside 27–32 kb positive sense single-stranded RNA genome (4, 5). Membrane glycoprotein (M), Nucleocapsid (N), Envelope (E), and Spike protein (S) are the crucial structural proteins of the virus while main protease and RNA-dependent RNA polymerase (RdRP) are the major non-structural proteins. Upon infection, the S glycoprotein binds to the human angiotensin converting enzyme-2 (ACE-2) receptor located mainly on the alveolar cells of the respiratory tract following the entry of the virus particles inside the host cells by the action of human transmembrane serine protease 2 (TMPRSS2) (6, 7). Moreover, the spike glycoprotein also interacts with the toll-like receptors 4 (TLR4) leading to the induction of strong proinflammatory responses in the lungs (8, 9). Induction of intense proinflammatory responses within the lungs termed “cytokine storm” is the principle cause of lung damage, multiple organ failure, and death (10, 11). Since its first report in the Wuhan province of China in December 2019, the SARS-CoV-2 has undergone a number of mutations, particularly in the S glycoprotein, resulting in the emergence of a number of variants (12) especially in UK, Europe, and India (13). Strains reported from India, namely, B.1.617 (Kappa), B.1.617.2 (Delta), and B.1.618, have been characterized as exceedingly transmissible SARS-CoV-2 variants (14). These variants possess mutations within the S protein that plays a major role in the viral infection through recognition of receptor and host cell membrane fusion (15). L452R, E484Q, D614G, and P681R mutations in the S protein have been documented in B.1.617 lineage while D145-146, E484K, and D614G mutations were prevalent in B.1.618 (14). Among these variants, the Delta strain possesses higher infectivity, mortality, and post-infection issues (16, 17). Mutant S proteins within these variants have been found to promote infectivity, transmission, and resistance to vaccine-induced immune response (18–20).

The clinical management strategy of COVID-19 primarily aims to alleviate the inflammation and the virus load. Recently, immunotherapies and antibody-based therapies targeting either the virus or virus-induced inflammation were also investigated (21–23). Several monoclonal antibodies (mAbs) like bamlanivimab, regdanvimab, tixagevimab, cilgavimab, etesevimab, casirivimab, imdevimab, and sotrovimab directed against the spike protein of SARS-CoV-2 to prevent the viral attachment and infection of host cell have been developed by several firms and are at various stages of clinical trials (24–26). These mAbs were developed against the wild-type SARS-CoV-2; however, emergence of variant forms of SARS-CoV-2 has raised questions on the efficacy of these mAbs against SARS-CoV-2 variants.

In this study, we have investigated the theoretical therapeutic efficacy of eight mAbs that are at various stages of development or clinical trials, against twenty SARS-CoV-2 variants of two different lineages of UK (B.1.1.7, Alpha) and Indian (B.1.617.2, Delta) origin having mutation in the S protein through *in silico* approaches. Furthermore, we also hypothesized a chimeric mAb for possible application against variant SARS-CoV-2 infection.

## Methods

### Data Mining

Mutated amino acids of spike glycoprotein of Alpha and Delta variants were retrieved from the GISAID database (https://www.gisaid.org/). Amino acid sequence of native spike glycoprotein (Accession ID: QHD43416.1) of SARS-CoV-2 and therapeutic mAbs were retrieved from the NCBI (https://www.ncbi.nlm.nih.gov/) and CoV-AbDab database (http://opig.stats.ox.ac.uk/webapps/covabdab/), respectively (27).

### Homology Modeling

Homology modeling is a template-dependent/independent method popularly used to model protein structure from its amino acid sequence. Based on the templates available in the database repository, an automated modeling server, SWISS-MODEL, was used to model all the mutant S proteins (28). Similarly, the web application ABodyBuilder, a tool for small-scale homology modeling (29), was applied for designing the Fv regions of the mAbs used in this study. The ABodyBuilder algorithm works through the following pathway, i.e., selection of template>prediction of orientation>prediction of the side chain>modeling of complementarity determining region loop to design computational model of the mAbs (30). Antibody i-Patch, a web tool that works on the antibody-specific statistics to determine the paratope and complementarity-determining regions (CDR) within the antibody, was also utilized to validate the efficacy of the paratopes towards an antigen (31). In addition, the webtool EpiPred was explored to determine the epitopes by using the homology model of the antibody as an input (32, 33). Each structure was verified using SAVES server that examines the stereochemical quality of a predicted protein structure through analyzing the residue-by-residue geometry and geometry of the overall structure (34, 35).

### Molecular Docking

The protein–protein interactions between the S protein variants and human mAbs were determined through molecular docking study using High Ambiguity Driven protein–protein DOCKing (HADDOCK) v. 2.4. This flexible docking program executes the docking process by using the information from known and/or predicted protein interfaces in ambiguous interaction restraints (AIR) and the outputs generated by this server were found to support experimentally validated (NMR and cryo-EM) structures of protein complexes (36). Herein, the PDB files of each SARS-CoV-2 variant and mAb were subjected to binding interactions in HADDOCK platform and the protein–protein interaction was predicted through a binding score provided by the webserver as well as analyzing the interacting residues within the output file. All the docked complexes were analyzed for biophysical interactions using Discovery Studio-2020 and PyMOL.

### Analyses of the Molecular Basis of Protein–Protein Interactions

Stability of the molecular topology, conformational topology, and dynamic behavior of the mAb–spike protein complex was studied through normal mode analyses (NMA) (37). Different protein complexes were analyzed for molecular flexibility and modal trajectories of structural dynamics using iMODS server from ChaconLab (38). NMA is a quantitative expression of motion of protein in a complex. In our study, NMA analyses were conducted to assess the biophysical attributes of S protein–mAb complexes. The iMODS server is a popular and customizable server that discloses coarse-grain (CG) levels and also determines the dihedral coordinates of C-alpha atoms in the proteins. iMods also delivers an NMA mobility that illustrates the collective motions and affine-model arrows that direct domain dynamics. Structural deformability usually depends on the helical content of a protein that is usually studied by analyzing the *B*-factor (39). Here, we studied the amplitude of atomic displacement in each spike protein–mAb complex by determining the *B*-factor. The relative impact of each deformation movement in the motion of interacting proteins within the S protein–mAb complex was studied by measuring the eigenvalues. Lower eigenvalues are indicative of greater stability while higher values reveal the reverse (4). The molecular flexibility, amplitude of fluctuation of each mode, and overall confirmational change in each S protein–mAb complex were studied by variance plot and covariance matrix. The docked complexes comprising S protein variants and mAbs were prepared in pdb format and submitted to the iMODS server to determine the normal modes within internal coordinates and details of the mobility (*B*-factor), structural deformability, covariance map, and linking matrix with eigenvalues. The data extracted from iMODS were analyzed and plotted using the statistical software package R. Changes in the conformation in S protein after binding of mAb were assessed by superimposing the unbound S protein structures on the S protein–mAb complex following our earlier reports (9, 39).

Selected variant S proteins of SARS-CoV-2 and the therapeutic mAbs were examined utilizing PRODIGY web tool to determine its binding affinity (Δ*G*) and dissociation constant (*K*_d_) (40).

### Designing of Chimeric Antibody

The rationale behind using *in silico* docking analysis was to check the binding efficacy of the mAbs against the S protein variants. The clear demonstration of the biomolecular interaction among S protein variants and mAbs was possible only through molecular docking. Hence, it was necessary to screen the possible efficacious mAbs and to obtain a precise picture on their respective CDRs to predict the possible interacting domains present in those antibodies. Such CDRs are important components of the chimeric antibody generated *in silico*.

After identifying the potential CDR patches in the screened mAbs, a series of chimeric antibodies were hypothesized for better efficacy. The binding domain of the mAbs were subjected to multiple sequence alignment and the peptide fragments showing binding affinity against SARS-CoV-2 variants were combined *in silico*. The resultant CDRs were fitted in a single amino acid chain to model the antibody structure by employing Therapeutic Antibody Profiler (TAP) (41, 42). The designed chimeric antibody was characterized for its immune-biological properties and efficacy against the S protein *in silico*.

## Results

This study seeks to determine the efficacy of various human mAbs reported to date for their binding to S proteins of SARS-CoV-2 Alpha and Delta variants.

### Screening of Efficacious mAbs Against Spike Protein Variants

For all the eight mAbs, 10 spike protein variants from Alpha and 10 from Delta lineages (shown in **Figure S1**) were tested for all possible interactions *in silico* (**Table S1**). A total of 160 mAb–spike protein complexes were obtained using Haddock 2.4 and were further screened by setting the docking score >100 as strong interaction while <50 as weak interaction (43). With these criteria, we studied 3 strong and 3 weak spike protein–mAb complexes for each mAb. The rationale behind such a selection was to filter the mAbs that are effective against a greater number of SARS-CoV-2 variants and also to understand the possible variants that could escape binding of the mAb. Regdanvimab, bamlanivimab, sotrovimab, etesevimab, and cilgavimab showed high docking scores against most of the Alpha and Delta variants (**Figure S2**). Particularly, regdanvimab displayed a high binding score against both Alpha and Delta variants. In contrast, tixagevimab, casirvimab, imdevimab, and cilgavimab possessed less docking scores. Docking score is a preliminary measurement of binding efficacy of the mAbs against the SARS-CoV-2 variants, and therefore, to validate the initial postulation as well as to obtain molecular insight on these interactions, we further refined our screening by including a total of 16 protein complexes comprising S protein variants from the Alpha lineage and 8 mAbs (**Table S1**). This was repeated for the Delta lineage.

### Comparative Analyses of the Biomolecular Interactions Between the mAbs and Spike Protein Variants and Studies on the Molecular Dynamics

The biophysical basis of mAb-SARS-CoV-2 spike protein interactions was investigated through determining the binding free energy and binding affinity. Eventually, three strongly bound and three weakly bound protein complexes were selected from each lineage (**Table 1**). P681H–tixagevimab, S982A–regdanvimab, and V70–cilgavimab complexes displayed significantly strong binding interactions with respective binding free energy (Δ*G*) of −15.1, −14.7, and −14.7 kcal/mol. Analysis of binding affinity in terms of the dissociation constant (*K*_d_) supported the inference of binding energy. The data revealed that P681H–tixagevimab, S982A–regdanvimab, and V70–cilgavimab possess *K*_d_ values of 7.8E−12, 1.6E−11, and 1.6E−11 respectively. These data suggested that tixagevimab, regdanvimab, and cilgavimab could efficiently bind to the SARS-CoV-2 variants of B.1.1.7 lineage. In contrast, weak interactions were noted for S982A–tixagevimab, T716I–cilgavimab, and S982A-etesevimab, indicating that the variants with spike protein mutations like S982A could escape the inhibitory effect of certain mAbs.

**Table 1:** Comparative analyses of the biophysical interactions (binding energy and binding affinity) between the mutated spike protein from SARS-CoV-2 variants and human mAbs.

Lineage of SARS-CoV-2 Strain	Spike protein with mutation	Bind with monoclonal antibody	Haddock score		Binding affinity Δ*G* (kcal/mol)	Dissociation constant *K*_d_ (M) at 25.0°C
B.1.1.7 (Alpha)	P681H	Bamlanivimab	Strongly docked	−104.8 ± 6.4	−11.9	1.9E−09
	D1118H		Weakly docked	−38.0 ± 10.3	−12.4	7.7E−10
	**S982A**	**Regdanvimab**	**Strongly docked**	−142.8 ± 4.1	**−14.7**	**1.6E−11**
	T716I		Weakly docked	−103.5 ± 6.8	−13.3	1.8E−10
	**P681H**	**Tixagevimab**	**Strongly docked**	−94.0 ± 7.6	**−15.1**	**7.8E−12**
	**S982A**		**Weakly docked**	−20.4 ± 6.5	**−11.1**	**6.7E−09**
	**V70-**	**Cilgavimab**	**Strongly docked**	−87.9 ± 7.5	**−14.7**	**1.6E−11**
	**T716I**		**Weakly docked**	−61.5 ± 6.1	**−8.7**	**4.2E−07**
	D614G	**Etesevimab**	Strongly docked	−139.0 ± 3.2	−10.9	1.1E−08
	**S982A**		**Weakly docked**	−51.0 ± 3.2	**−12.2**	**1.1E−09**
	H69-	Casirivimab	Strongly docked	−72.5 ± 5.0	−13.8	7.5E−11
	Y144-		Weakly docked	−33.3 ± 1.9	−13.1	2.6E−10
	V70-	Imdevimab	Strongly docked	−76.6 ± 5.1	−9.9	5.3E−08
	T716I		Weakly docked	−34.9 ± 11.8	−14.4	2.8E−11
	D1118H	Sotrovimab	Strongly docked	−132.2 ± 15.4	−14.1	4.6E−11
	D614		Weakly docked	−67.1 ± 14.4	−13.0	2.8E−10
B.1.617.2 (Delta)	**P681R**	**Bamlanivimab**	**Strongly docked**	−106.3 ± 5.4	**−14.1**	**4.7E−11**
	**T19R**		**Weakly docked**	−50.0 ± 13.3	**−10.0**	**4.4E−08**
	D614G	**Regdanvimab**	Strongly docked	−140.3 ± 3.1	−10.7	1.4E−08
	**T19R**		**Weakly docked**	−117.8 ± 10.6	**−10.6**	**1.6E−08**
	**P681R**	**Tixagevimab**	**Strongly docked**	−90.4 ± 4.0	**−13.9**	**6.9E−11**
	**G142D**		**Weakly docked**	−22.6 ± 10.3	**−10.2**	**3.1E−08**
	E156G	Cilgavimab	Strongly docked	−112.2 ± 11.4	−12.4	8.1E−10
	D614G		Weakly docked	−66.1 ± 17.2	−11.5	3.5E−09
	D614G	Etesevimab	Strongly docked	−139.0 ± 3.2	−10.9	1.1E−08
	R158-		Weakly docked	−38.9 ± 18.9	−13.4	1.6E−10
	T478K	Casirivimab	Strongly docked	−74.9 ± 5.0	−12.0	1.5E−09
	G142D		Weakly docked	−37.3 ± 9.3	−12.9	3.5E−10
	P681R	Imdevimab	Strongly docked	−82.6 ± 5.3	−13.1	2.3E−10
	T19R		Weakly docked	−37.7 ± 16.9	−11.0	7.9E−09
	**R158-**	**Sotrovimab**	**Strongly docked**	−132.0 ± 7.9	**−13.3**	**1.6E−10**
	D614G		Weakly docked	−67.1 ± 14.4	−13.0	2.8E−10

Bold values are depicting the 3 strongly and 3 weakly docked structure.

For B.1.617.2 lineage, P681R–bamlanivimab, P681R–tixagevimab, and R158–sotrovimab complexes revealed strong interactions with respective binding free energies (Δ*G*) of −14.1, −13.9, and −13.3 kcal/mol. Furthermore, SARS-CoV-2 that harbors S protein mutations T19R and G142D were predicted as the variants that could escape from the inhibitory effects of aforesaid mAbs.

Taking clues from earlier studies, the top three strongly bound and three weakly bound mAb–spike protein variant complexes from each lineage were studied for protein–protein interactions at the molecular level (**Table 1**). The topological differences in binding pattern of different mAbs to S protein variants as well as involvement of various non-covalent bonds/forces are summarized in **Figure S3** and **Table 2**. While studying the most strongly bound complex for the B.1.1.7 lineage, the P681H–tixagevimab complex was found to be stabilized by the most number (a total of 26 H-bonds) of hydrogen bonds. In addition, hydrophobic interaction (π–Alkyl: 1) was found to provide additional stability in holding the two proteins (**Figure S3A** and **Table 2**). The other strongly bound complexes, viz., S982A–regdanvimab and V70–cilgavimab, were also found to possess similar numbers of H-bonds. However, additional interactive forces (4 hydrophobic interactions; π–Alkyl: 3 and alkyl: 1; and electrostatic bonds: 2) were noted in V70–cilgavimab (**Figure S3B** and **Table 2**). However, the topology of binding was dissimilar (as evident from the interacting amino acids) to that of tixagevimab and that could explain the reduced binding affinity. In comparison to the high-affinity antibodies, we found lesser degree of interactions in terms of hydrogen bonding and/or other noncovalent interactions leading to weak binding of mAb to S variants as evident for T716I–cilgavimab, S982A–tixagevimab, and S982A-etesevimab (**Figures S3A, B** and **Table S3**).

**Table 2:** Biomolecular interactions amongst the spike proteins and high affinity monoclonal antibodies.

Interaction details											
Antigen residues	Fab residues	Matched with SAbPred predicted CDR residues	Distance (Å)	Antigen residues	Fab residues	Matched with SAbPred predicted CDR residues	Distance (Å)	Antigen residues	Fab residues	Matched with SAbPred predicted CDR residues	Distance (Å)
**B.1.1.7 (Alpha) -Lineage of SARS-CoV-2 Strain**											
**P681H–tixagevimab**				**S982A–regdanvimab**				**V70–cilgavimab**			
**Hydrogen Bond**											
GLY472	ARG44		1.846	ASP3092	LYS66		1.57546	ASP2610	LEU109	Y	1.68776
SER1561	ASN57	Y	2.20629	ASP2536	LYS73		2.66857	TYR1565	LYS150	Y	2.38416
TYR436	ARG67		3.07872	LYS2527	ASP56	Y	3.21068	SER2617	SER154	Y	1.79876
VAL1485	GLU122		2.11712	LYS2527	ASP56	Y	1.56226	GLU2586	TYR157	Y	1.73972
VAL1485	GLN148	Y	2.99581	ASN2852	GLY33	Y	2.24497	GLU2586	SER159	Y	1.74693
VAL1485	GLN148	Y	2.88623	ASN2529	LYS59		1.70743	GLY1601	LYS162	Y	2.56925
CYS1454	SER149	Y	2.03091	GLU2530	HIS61		2.14448	PHE1602	LYS162	Y	1.95827
GLU1458	SER151	Y	1.74429	ASN1674	THR67		1.87769	GLY1601	THR185	Y	1.82372
ALA1462	SER215	Y	1.80752	GLN749	TYR105	Y	2.44674	GLN1609	SER199		2.6446
SER1489	ARG217	Y	2.63386	ASP3079	TYR107	Y	2.91451	PHE2584	SER159	Y	2.89539
SER1489	ARG217	Y	1.73988	LYS3074	TYR155	Y	2.47154	GLY2585	SER159	Y	1.78483
ASN435	GLU66		2.6411	ASN3073	ASN174	Y	2.37936	ASN2589	TYR224	Y	2.07595
GLN480	GLN62		2.88996	SER3189	LYS176	Y	2.19465	ASN2616	SER199		1.97765
PHE484	GLU66		1.83268	ASN3073	LYS189		1.98564	LYS2632	PRO108	Y	2.21217
ASN488	GLU66		3.0399	ASP3069	GLY191		2.45689	GLY1601	SER184	Y	3.01655
GLY1457	SER149	Y	2.53091	LYS2527	ASN58	Y	1.92671	GLY1612	GLU202		3.33806
ASN1461	GLY214	Y	2.5667	LYS2527	ASP56	Y	3.08543	THR2631	PRO108	Y	3.25262
ASN1461	ARG217	Y	2.01651	THR2535	ASP57	Y	1.62597				
THR1463	TYR154	Y	1.85134	ASN2852	GLY33	Y	2.40857				
ARG1464	SER216	Y	2.74405	ASN3073	LYS189		2.52172				
ASN1488	GLU122		1.77181	LEU3077	ASN153	Y	2.01706				
SER1489	GLU122		1.84294	ALA3078	ASN153	Y	2.00835				
LEU1453	SER149	Y	3.22639	GLN3198	ASN106	Y	2.43996				
GLU1458	SER151	Y	3.29199	SER3188	LYS176	Y	3.40308				
ASN1461	SER215	Y	3.02321	ASP3069	SER190		3.14642				
GLY434	GLU66		3.41544	SER3188	ASN175	Y	3.32419				
PRO1455	SER149	Y	2.95993	GLN3259	TYR105	Y	3.59512				
**Electrostatic Bond**											
				GLU2530	LYS59		4.65638				
				LYS3074	ASP173	Y	4.51837				
**Hydrophobic Bond (π–Alkyl)**											
TYR436	ARG67		5.06439	ALA753	TYR105	Y	4.49149				
				VAL995	TYR105	Y	5.34089				
				LEU999	TYR105	Y	5.42826				
**Hydrophobic Bond (Alkyl)**											
				ALA3094	LYS66		4.76062				
**Hydrophobic Bond (π- π)**											
								PHE1602	TRP182	Y	5.46997

**B.1.617.2 (Delta)-Lineage of SARS-CoV-2 Strain**											
**P681R–bamlanivimab**				**P681R–tixagevimab**				**R158–sotrovimab**			
**Hydrogen Bond**											
GLU327	ARG50		1.55363	GLY2734	ARG44		2.24393	ASP1955	ARG141		2.77667
GLU327	ARG50		2.39339	SER430	ASN57	Y	2.58051	ALA1804	THR74	Y	3.03559
GLY326	ARG50		2.56527	TYR2698	ARG67		2.59736	ALA3012	TRP104	Y	2.57857
ASP351	HIS104	Y	1.69139	VAL354	GLN148	Y	2.61321	GLN2118	SER106	Y	2.67731
GLU327	TYR106	Y	1.75457	CYS323	SER149	Y	2.2566	THR2122	SER106	Y	1.89863
GLU2733	SER178	Y	2.35228	GLU327	SER151	Y	1.7445	GLU1397	SER154	Y	2.77409
CYS323	ALA103	Y	2.90251	ASN330	SER215	Y	1.99254	ASN1940	ARG169		1.84746
PHE325	TYR106	Y	2.80316	SER358	ARG217	Y	2.25764	ALA1945	ARG178	Y	1.6848
GLY326	TYR106	Y	1.86565	PHE325	SER149	Y	3.07747	ALA1945	ARG178	Y	2.44364
GLN2747	SER152	Y	2.014	GLY326	SER149	Y	2.37639	THR1943	THR180	Y	1.79555
ASN2750	TYR214	Y	2.35522	ASN330	ARG217	Y	1.68917	VAL1942	GLY181		2.30551
SER358	TYR105	Y	3.23675	THR332	TYR154	Y	2.34097	ASP1959	GLY190		1.69436
GLN2747	SER150	Y	3.24228	ARG333	SER216	Y	2.09294	THR1720	SER31	Y	1.80176
TYR2698	SER152	Y	3.42903	ASN357	GLU122		1.83192	GLN1723	TYR54	Y	2.02886
GLY2695	GLY190		3.73547	SER358	GLU122		3.08345	GLN2065	GLY103	Y	1.89888
GLY2745	SER152	Y	3.47513	VAL432	ASN57	Y	2.96427	ASN2069	ARG102	Y	2.14298
				TYR2698	GLN65		2.69082	GLN3008	SER106	Y	2.1518
				TYR2698	GLU66		1.70023	PHE1939	GLY181		3.64089
				ASN2750	GLU66		3.06276	THR1720	THR30	Y	3.11702
				ASN2750	GLU66		2.85583	SER2119	SER106	Y	3.10337
				LEU322	SER149	Y	3.40152	ARG1963	SER176		1.98702
				ASN330	SER215	Y	2.94661				
				PRO324	SER149	Y	2.94922				
				GLY2745	GLU66		3.14184				
**Electrostatic Bond**											
GLU327	ARG50		5.30159					ASP1955	ARG141		5.484
								ASP1964	ARG178	Y	4.58879
								LYS1927	GLU205		5.0261
**Hydrophobic Bond (π–σ)**											
VAL354	HIS104	Y	3.69105								
SER358	TYR105	Y	3.50298								
**Hydrophobic Bond (π–π)**											
PHE2735	TYR100	Y	5.22663								
**Hydrophobic Bond (Alkyl)**											
LEU322	ALA103	Y	4.80066					PRO1928	PRO204		4.27859
								ALA1945	ARG178	Y	4.16232
								ALA2058	VAL2		4.96639
**Hydrophobic Bond (π–Alkyl)**											
VAL354	TYR101	Y	4.57784	TYR2698	ARG67		5.21088	ALA3012	TRP104	Y	5.08227
VAL354	TYR105	Y	5.04138					ALA3258	TRP104	Y	5.01111
								ALA3012	TRP104	Y	5.48531
								PHE1939	PRO183		5.48537

Biomolecular interactions between mAbs and S protein variants were also studied for the B.1.617.2 lineage. P681R–bamlanivimab, P681R–tixagevimab, and R158–sotrovimab displayed a strong binding as evidenced from their respective binding free energy (**Table 1**, **Figures S3C, D** and **Tables 2** and **S3**). Considering the topology of binding (**Figure S3C**), bamlanivimab exhibited a very strong binding with S protein variant P681R, and this binding was stabilized by 16 H bonding, 1 electrostatic interaction, and 6 hydrophobic interactions (π–σ: 2; π–π: 1; π–Alkyl: 2 and alkyl: 1). Interestingly, tixagevimab was also found to interact with the same variant with a similar binding topology but with less affinity than that of bamlanivimab (**Figure S3C**). The abundance of H-bonds was also similar, and hence, lesser number of hydrophobic interactions could be responsible for the differences in the avidity of tixagevimab and bamlanivimab, while sotrovimab possessed strong affinity to the R158 variant and this interaction was found to be stabilized by non-covalent forces including H-bonding (**Table 2**). Weak interactions were observed for T19R–bamlanivimab, G142D–tixagevimab, and T19R–regdanvimab (**Figure S3D** and **Table S3**).

We scrutinized the conformational changes in S1 protein following its binding to mAb. Changes in the conformation of heterotrimeric S1 protein after binding of an antibody is key for the neutralization of SARS-CoV-2 (44). We observed clear changes in the conformation of S protein variants following interaction with the strongly binding mAbs (**Figure S4A**). This observation supports the earlier data that demonstrated that tixagevimab is the strongest neutralizing mAb against SARS-CoV-2 variants. However, binding of cilgavimab to T716I revealed no sign of conformational changes and indicated that T716I could evade cilgavimab (**Figure S4B**). The mode of binding of mAbs and subsequent changes in the conformation were also verified by comparing tixagevimab–P681H and cilgavimab–T716I protein complexes as well as a superimposed form of them (**Figure S4C**).

We have also studied the molecular dynamics of S protein–mAb complexes. The comparative analyses of molecular dynamics of the most stable S protein–mAb complex, i.e., P681H–tixagevimab, and the weakest bound complex, i.e., T716I–cilgavimab, revealed significant differences in various intramolecular and intermolecular parameters (**Figure S5**). Clear difference in the direction of molecular motion was observed between the two protein complexes (**Figures S5A–F**). A relatively lower level of deformability described the compactness of P681H–tixagevimab while the reverse was observed for T716I–cilgavimab. Furthermore, protein components in the T716I–cilgavimab complex had higher mobility, indicating weak association, while the P681H–tixagevimab complex had lesser mobility, thus denoting the strong association between components of the complex (**Figures S5E, F**). Eigenvalue is a measure of deformation due to fluctuation in protein motion (38). A high eigenvalue is indicative of a localized displacement while low eigenvalue indicates cumulative conformational changes in the protein structure (38, 39). A low eigenvalue of 3.407305e^-05^ for P681H–tixagevimab revealed lower energy deformation of the structure resulting in greater stability of the complex and conformational changes in the S protein after forming complex with mAb (**Figure S5G**). In contrast, T716I–cilgavimab displayed the reverse characteristics and interpreted as relatively unstable complex.

Structural integrity of the S protein–mAb was also investigated by molecular flexibility by measuring the theoretical fluctuation (variance) of each mode in a protein complex (37). A higher degree of flexibility was observed for P681H–tixagevimab, suggesting a higher affinity of two proteins towards each other while the reverse was observed for T716I–cilgavimab (**Figures S5I, J**). Covariance maps demonstrated abundance of correlated motions of Cα atoms in P681H–tixagevimab, thus indicating motion stiffness and rigidity in the protein complex compared to T716I–cilgavimab (**Figures S5K, L**).

### Designing of Chimeric mAb as Potential Broad-Spectrum Immunotherapeutics

**Figure 1:**
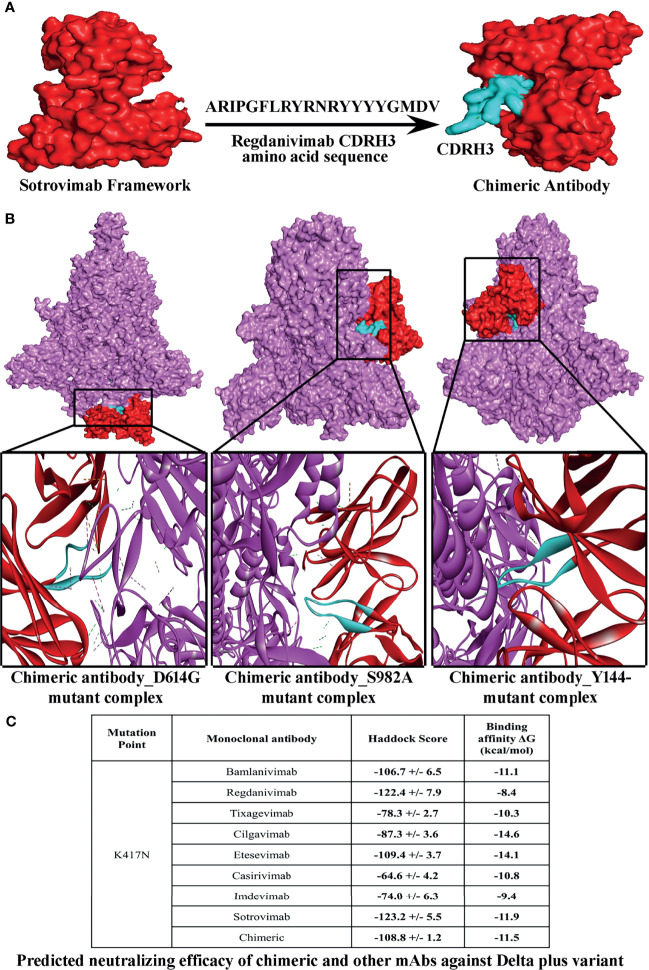
Structure of a chimeric mAb for inducing high-affinity binding against the greatest number of SARS-CoV-2 spike protein variants. **(A)** Depicts steps involved in the conception of a chimeric mAb. **(B)** Visualization of interactions of chimeric mAb with 3 variants of SARS-CoV-2 spike protein, namely, D614G, S982A, and Y144-. **(C)** Predicted neutralizing efficacy of chimeric and other mAbs against Delta plus variant.

Our *in silico* analyses predicted that a number of S protein variants depict very weak binding to mAbs, and therefore, these mAbs might not be effective against newly emerged strains especially Delta plus strain (B.1.617.2.1). This has prompted us to design a chimeric mAb that could be effective in encountering most of the variants (**Table S3**). We have combined the CDRs of the strongly interactive mAb to prepare a series of chimeric mAbs and tested against the spike protein variants (**Tables S3** and **S4A, B**). Eventually, when we incorporated CDRH3 of regdanvimab (ARIPGFLRYRNRYYYYGMDV) within the framework of sotrovimab without hampering its CDR (ARDYTRGAWFGESLIGGFDN), the resultant paratope was found to display very strong binding with most of the escape variants from both lineages (**Figures 1A–C** and **Table S5**). Even the physico-biochemical properties, like solubility and hydropathicity of the selected chimeric mAb, supported its durability in neutralizing the variants (**Table S6**). When we compared the efficacy of this mAb against the newly found Delta plus strain (B.1.617.2.1) and Beta strain (B.1.351), a very satisfactory binding interaction was observed as compared to the eight therapeutic mAbs (**Figure 1C** and **Table S7**). However, binding efficacy of the chimeric antibody against Gamma strain (P.1) was comparatively lower than that of the Delta, Delta plus, and Beta strain (**Figure 1C**, **Tables S5** and **S7**).

## Discussion

Within a year of its outbreak, SARS-CoV-2 has undergone various mutations leading to the emergence of a number of variants with altered antigenicity and virulence. The B.1.1.7 lineage constituting the Alpha variants was first detected in UK in September 2020 and later spread to as many as 50 countries including India (45). The B.1.617 lineage was first detected in India in March 2021, and this lineage constitutes three subtypes, viz., B.1.617.1, B.1.617.2, and B.1.617.3 (46). The B.1.617.2 sub-lineage is the most virulent strain and named as Delta variant (46). High transmission rate has been the major trait of Alpha strains while Indian strains especially the Delta strains are characterized by high infectivity, severity of infection, and mortality.

S protein is the major pathogenic protein of SARS-CoV-2. In addition to attachment and infection, the S protein–TLR4 interaction is known to promote macrophage activation syndrome, acute respiratory distress syndrome, and cytokine storm leading to multi-organ damage (44, 47). Therefore, S protein is currently the major target for developing various prophylaxis and therapeutic strategies. Interestingly, several mAbs have been developed to block S protein interaction with the host receptor and to prevent the infection. Recent reports have depicted the success of some of these human mAbs in combating COVID-19 (48–50). Clinical trials supported the emergency use authorization (EUA) of bamlanivimab for treating COVID-19 patients (Coronavirus (COVID-19) Update, 2021). However, this EUA has been revoked by the Food and Drug Administration (FDA) due to the emergence of bamlanivimab resistance (51). Bamlanivimab in combination with etesevimab was reported to cause a sharp decline in the viral load as well as immunopathological consequences (21, 51). Currently bamlanivimab and etesevimab are considered as investigational drugs and are yet to receive EUA (21). Casirivimab–imdevimab combinational therapy also received EUA and clinical trials revealed satisfactory reduction in the viral load and severity of lung disease after treatment (21). This mAb combination induces phagocytosis of the infected cells through antibody-mediated cytotoxicity (11). EUA-approved sotrovimab targets the highly conserved epitope of the receptor binding domain (RBD) of the S spike protein and blocks the attachment of S protein to ACE2 (52). While regdanivimab displays strong neutralizing activity against the Delta variant with 100% survival rate in pre-clinical studies, phase III clinical data revealed reduction in the COVID-19-related hospitalization, death, and/or reduction in the recovery time of high-risk patients (53–55).

Our study presents a comparative efficacy of eight human mAbs for their binding to RBD of S glycoprotein from Alpha and Delta variants of SARS-CoV-2. Newly emerged strains have distinct host–virus interaction properties due to the occurrence of mutations in the RBD of S protein (6). Our initial binding analyses indicated that regdanvimab, bamlanivimab, sotrovimab, etesevimab, and cilgavimab could be considered for treating both Alpha and Delta strains. While comparing the response, we found that Delta strains are more responsive to the mAbs. This is a very interesting finding as most of the Delta variants reported so far could evade either wild-type SARS-CoV-2 infection-induced or vaccine-induced antibodies (14, 16). Our *in silico* studies collectively suggested that tixagevimab, regdanvimab, and cilgavimab could be the therapeutic choices for Alpha strains (B.1.1.7), while bamlanivimab, tixagevimab, and sotrovimab could be used for treating the Delta variants (B.1.617.2). Considering the *in silico* data against both B.1.1.7 and B.1.617.2 lineages, tixagevimab has been predicted to be the most potential mAb.

Conformational change is an important parameter in antigen–antibody interaction (56). We documented the changes in S protein conformation after binding of tixagevimab, and this change in configuration was found stable after analyzing the protein motion through molecular dynamics of the tixagevimab–S protein complex. Such a stable conformational change was absent in the S protein of the escape strains. In fact, a large number of both Alpha and Delta strains were found to evade the mAbs. Therefore, we attempted to develop a chimeric antibody that could neutralize the escaping strains of both Alpha and Delta lineages including the very recently emerged Delta plus strain. A chimeric antibody prepared using the framework of sotrovimab and the CDRH3 of regdanivimab could provide very efficient binding against most of the escape strains of Alpha, Delta, and Delta plus strains. Moreover, the binding efficiency of the chimeric antibody was found to be higher than each constituent mAb and the other six mAbs included in this study. Therefore, transformation of these predictive lines of evidence to further application level through experimental validation is expected to provide an excellent strategy to combat the emerging lethal strains of SARS-CoV-2.

In this *in silico* study, we have used several human therapeutic mAbs that were developed for treating COVID-19 (22, 57). Clinical trials of Celltrion’s regdanivimab and sotrovimab from GSK and Vir Biotechnology showed their efficacy in neutralizing the SARS-CoV-2 variants of B.1.617 and B.1.1.7 lineages (58). Therefore, combining both mAbs to generate a chimeric antibody could be an attractive option to target newly emerging SARS-CoV-2 variants. In this context, rationally designed chimeric antibodies comprising an IgG1 framework with ACE2 units grafted on the CDR patches have been designed for efficient binding and neutralization of SARS-COV-2 variants. In addition, a fusion protein called ACE2-Ig by connecting the extracellular domain of ACE2 to the Fc region human IgG1 has also been designed. This fusion protein was experimentally validated under *in vitro* conditions and found to exhibit a high degree of cross-reactivity against SARS-CoV and SARS-CoV-2 (59, 60).

Conception of therapeutic strategies *via in silico* approaches, especially identification of antigenic proteins/epitopes, designing of antibodies and vaccines, and development of new drugs and drug targets are in the current trends. To date, immunoinformatics and computational structural biology have been successfully implicated in engineering a number of efficacious immunotherapeutic agents including vaccines, chimeric antibodies, and mAbs for treating various infectious and inflammatory diseases of human (61–74). In this context, a theoretical design study by Pelat et al. (75) has demonstrated germ-line humanization of a non-human primate antibody fragment, namely, Fab 35PA_83_, which neutralizes anthrax toxin. Moreover, a study by Wolf Pérez et al. (76) strongly campaigns for the acceptability of *in silico*-based design and manipulation of therapeutic human mAbs. In fact, by using an *in silico* solubility predictor tool, CamSol, the authors designed 17 variants of a humanized mAb (IgG4) and found a robust correlation between the values predicted *in silico* and obtained by experiments (76). To date, a number of *in silico* studies have been conducted to understand the basic biology of SARS-CoV-2 as well as to propose new intervention strategies against COVID-19. Interestingly, many of them have been validated successfully, which prompted us to imply a similar strategy to screen for the efficacy of various anti-S human mAbs towards SARS-CoV-2 variants and to design a chimeric antibody. Our *in silico* theoretical findings on chimeric mAb has revealed a high degree of efficacy against the newly emerged SARS-CoV-2 variants, and we expect that this chimeric antibody might exert similar functions *in vitro* and *in vivo*.

## Conclusion

Both monoclonal and polyclonal antibodies directed against SARS-CoV-2 structural proteins or inflammatory mediators have been employed for treating COVID-19 patients (77). The present study adds a new dimension to the existing knowledge on the efficacy of anti-S protein human mAbs against COVID-19 based on the hypothesis that mAbs could directly bind to the mutant spike proteins of the Alpha and Delta strains of SARS-CoV-2 to neutralize them. Among the 8 human mAbs so far tested against SARS-CoV-2, our *in silico* evidence suggests that tixagevimab, regdanvimab, and cilgavimab could efficiently neutralize most of the B.1.1.7 strains while bamlanivimab, tixagevimab, and sotrovimab could effectively inhibit the Delta variants. Moreover, the concept of chimeric mAb could also be taken into consideration for the treatment of COVID-19 patients infected with the newly emerged strains. However, the limitation of our study is that our findings are theoretical and have not been tested experimentally. Therefore, we welcome further experimental investigation to validate our conclusions, and this will indeed put forward the promising implication of mAb/chimeric mAb-based immunotherapies to combat newly emerging SARS-CoV-2 variants.

## Data Availability Statement

The original contributions presented in the study are included in the article/**Supplementary Material**. Further inquiries can be directed to the corresponding authors.

## Author Contributions

Performed the experiments: ND and PC. Analyzed the data: ND, PC, JB, and SM. Wrote the manuscript: JB and SM. Conceived, designed, and supervised the study: JB and SM. All authors contributed to the article and approved the submitted version.

## Conflict of Interest

The authors declare that the research was conducted in the absence of any commercial or financial relationships that could be construed as a potential conflict of interest.

## Publisher’s Note

All claims expressed in this article are solely those of the authors and do not necessarily represent those of their affiliated organizations, or those of the publisher, the editors and the reviewers. Any product that may be evaluated in this article, or claim that may be made by its manufacturer, is not guaranteed or endorsed by the publisher.
