# Supplementary material for: In Silico Analyses on the Comparative Potential of Therapeutic Human Monoclonal Antibodies Against Newly Emerged SARS-CoV-2 Variants Bearing Mutant Spike Protein
Source: Front Immunol. 2022 Jan 10;12:782506. doi: 10.3389/fimmu.2021.782506 (PMC8784557; doi:10.3389/fimmu.2021.782506)
Supplement: Supplementary file 1 [file DataSheet_1.zip › Final revised supplementary material.docx]

***Supplementary Materials***

***In silico* analyses on the comparative potential of therapeutic human monoclonal antibodies against newly emerged SARS-CoV-2 variants bearing mutant spike protein**

**Running head: *Monoclonal Antibodies as Anti-SARS-CoV-2 Therapeutics***

Nabarun Chandra Das^1#^, Pritha Chakraborty^1#^, Jagadeesh Bayry^2,*^ and Suprabhat Mukherjee^1,*^

^1^Integrative Biochemistry & Immunology Laboratory, Department of Animal Science,

Kazi Nazrul University, Asansol-713 340, West Bengal, India; nabarunchandradas@gmail.com (N.C.D), prithachakraborty895@gmail.com (P.C), suprabhat.mukherjee@knu.ac.in (S.M)

^2^ Department of Biological Sciences & Engineering, Indian Institute of Technology Palakkad, Palakkad, 678 623 India

^#^Contributed equally.

**^*^Corresponding authors.** J. Bayry: [bayry@iitpkd.ac.in](mailto:bayry@iitpkd.ac.in) and S. Mukherjee: [suprabhat.mukherjee@knu.ac.in](mailto:suprabhat.mukherjee@knu.ac.in);[babaimbc@gmail.com](mailto:babaimbc@gmail.com)

**Table S1:** Molecular docking-based screening of the efficacious mAb against the mutant spike protein from the Alpha and Delta variants of SARS-CoV-2.

| **Sl. No.** | **Lineage of SARS-CoV-2 Strain** | **Spike protein with mutation** | **Prevalence (India)** | **Prevalence (U.K.)** | **Interacting Monoclonal Antibody** | **Binding Energy** | **Haddock 2.4 score^$$^** |
| --- | --- | --- | --- | --- | --- | --- | --- |
|  |  |  |  |  |  |  |  |
| 1. | B.1.1.7  (Alpha) | A570D  D614G  **D1118H**  H69-  N501Y  **P681H**  S982A  T716I  V70-  Y144- | 98.31%  99.35%  96.75%  53.97%  94.41%  98.7%  99.09%  98.83%  53.84%  61.90% | 99.96%  99.98%  99.89%  99.52%  98.93%  99.91%  99.96%  99.93%  99.42%  98.84% | Bamlanivimab | -170.663  -90.9341  -46.8991  -109.76  -45.3444  -126.204  -12.5998  -104.297  -129.976  -28.8848 | -44.4 +/- 16.1  -92.5 +/- 6.6  **-38.0 +/- 10.3**  -54.1 +/- 14.3  -91.8 +/- 6.8  **-104.8 +/- 6.4**  -48.1 +/- 5.1  -49.3 +/- 4.9  -87.6 +/- 5.1  -54.0 +/- 7.4 |
| 2. |  | A570D  D614G  D1118H  H69-  N501Y  P681H  **S982A**  **T716I**  V70-  Y144- | 98.31%  99.35%  96.75%  53.97%  94.41%  98.7%  99.09%  98.83%  53.84%  61.90% | 99.96%  99.98%  99.89%  99.52%  98.93%  99.91%  99.96%  99.93%  99.42%  98.84% | Regdanvimab | -292.515  -109.15  -251.174  -229.863  -268.5  -138.38  -309.24  -243.177  -134.727  -186.222 | -126.1 +/- 20.4  -140.3 +/- 3.1  -124.7 +/- 7.7  -142.3 +/- 12.8  -121.3 +/- 8.0  -137.2 +/- 8.1  **-142.8 +/- 4.1**  **-103.5 +/- 6.8**  -129.4 +/- 6.0  -120.7 +/- 6.6 |
| 3. |  | A570D  D614G  D1118H  H69-  N501Y  **P681H**  **S982A**  T716I  V70-  Y144- | 98.31%  99.35%  96.75%  53.97%  94.41%  98.7%  99.09%  98.83%  53.84%  61.90% | 99.96%  99.98%  99.89%  99.52%  98.93%  99.91%  99.96%  99.93%  99.42%  98.84% | Tixagevimab | -208.111  -6.46754  -71.0924  -74.422  -76.4304  -158.603  -130.292  -63.6636  -102.785  -213.332 | -35.3 +/- 11.9  -72.1 +/- 5.6  -21.5 +/- 10.8  -80.1 +/- 4.7  -37.6 +/- 3.3  **-94.0 +/- 7.6**  **-20.4 +/- 6.5**  -30.6 +/- 13.6  -78.8 +/- 2.2  -30.4 +/- 13.4 |
| 4. |  | A570D  D614G  D1118H  H69-  N501Y  P681H  S982A  **T716I**  **V70-**  Y144- | 98.31%  99.35%  96.75%  53.97%  94.41%  98.7%  99.09%  98.83%  53.84%  61.90% | 99.96%  99.98%  99.89%  99.52%  98.93%  99.91%  99.96%  99.93%  99.42%  98.84% | Cilgavimab | -142.908  -148.188  -51.5191  -140.321  -113.573  -5.74673  -139.123  -94.5386  -115.301  -116.482 | -73.6 +/- 18.9  -66.1 +/- 17.2  -82.2 +/- 4.8  -77.6 +/- 3.6  -83.4 +/- 9.9  -76.9 +/- 8.1  -82.2 +/- 7.3  **-61.5 +/- 6.1**  **-87.9 +/- 7.5**  -76.6 +/- 4.8 |
| 5. |  | A570D  **D614G**  D1118H  H69-  N501Y  P681H  **S982A**  T716I  V70-  Y144- | 98.31%  99.35%  96.75%  53.97%  94.41%  98.7%  99.09%  98.83%  53.84%  61.90% | 99.96%  99.98%  99.89%  99.52%  98.93%  99.91%  99.96%  99.93%  99.42%  98.84% | Etesevimab | -145.335  -113.256  -179.36  -96.5263  -227.191  -86.2948  -96.0295  -146.696  -47.8196  -136.956 | -60.9 +/- 13.7  **-139.0 +/- 3.2**  -59.1 +/- 13.0  -106.1 +/- 6.0  -58.1 +/- 13.8  -113.1 +/- 2.3  **-51.0 +/- 3.2**  -52.5 +/- 13.2  -113.3 +/- 3.1  -60.7 +/- 3.0 |
| 6. |  | A570D  D614G  D1118H  **H69-**  N501Y  P681H  S982A  T716I  V70-  **Y144-** | 98.31%  99.35%  96.75%  53.97%  94.41%  98.7%  99.09%  98.83%  53.84%  61.90% | 99.96%  99.98%  99.89%  99.52%  98.93%  99.91%  99.96%  99.93%  99.42%  98.84% | Casirivimab | -71.7517  -20.9452  -73.0985  -112.836  -115.268  -40.5912  -68.3445  -195.341  -43.3942  -69.5715 | -50.8 +/- 12.0  -59.7 +/- 4.4  -46.1 +/- 12.7  **-72.5 +/- 5.0**  -43.8 +/- 5.1  -71.0 +/- 1.9  -41.0 +/- 9.7  -40.4 +/- 11.5  -68.5 +/- 1.4  **-33.3 +/- 1.9** |
| 7. |  | A570D  D614G  D1118H  H69-  N501Y  P681H  S982A  **T716I**  **V70-**  Y144- | 98.31%  99.35%  96.75%  53.97%  94.41%  98.7%  99.09%  98.83%  53.84%  61.90% | 99.96%  99.98%  99.89%  99.52%  98.93%  99.91%  99.96%  99.93%  99.42%  98.84% | Imdevimab | -143.279  -3.93831  -174.166  -96.2476  -140.146  -90.7257  -79.1173  -171.546  -76.4667  -187.938 | -35.4 +/- 7.8  -65.4 +/- 5.0  -41.6 +/- 15.3  -70.0 +/- 1.2  -42.7 +/- 9.8  -35.0 +/- 8.3  -76.3 +/- 4.8  **-34.9 +/- 11.8**  **-76.6 +/- 5.1**  -42.3 +/- 6.1 |
| 8. |  | A570D  **D614G**  **D1118H**  H69-  N501Y  P681H  S982A  T716I  V70-  Y144- | 98.31%  99.35%  96.75%  53.97%  94.41%  98.7%  99.09%  98.83%  53.84%  61.90% | 99.96%  99.98%  99.89%  99.52%  98.93%  99.91%  99.96%  99.93%  99.42%  98.84% | Sotrovimab | -168.066  -117.134  -196.776  -174.453  -180.051  -148.794  -118.545  -150.351  -152.269  -282.877 | -115.5 +/- 8.2  **-67.1 +/- 14.4**  **-132.2 +/- 15.4**  -122.0 +/- 5.0  -112.3 +/- 2.7  -129.0 +/- 14.7  -115.9 +/- 7.4  -122.7 +/- 5.4  -113.1 +/- 6.0  -124.3 +/- 6.3 |
| 9. | B.1.617.2  (Delta) | D950N  E156G  F157-  G142D  L452R  **P681R**  R158-  **T19R**  T478K  D614G | 84.45%  32.51%  32.51%  42.93%  97.17%  97.44%  32.51%  94.96%  97.17%  99.82% | 99.49%  98.87%  98.89%  62.02%  99.75%  100.00%  98.87%  99.47%  99.75%  100.00% | Bamlanivimab | -128.829  -62.7746  -51.8175  -113.518  -91.9380  -106.45  -101.896  -61.8244  -94.5644  -90.9341 | -53.5 +/- 9.4  -59.4 +/- 2.3  -91.0 +/- 3.3  -57.7 +/- 9.8  -103.6 +/- 4.1  **-106.3 +/- 5.4**  -55.1 +/- 4.9  **-50.0 +/- 13.3**  -105.0 +/- 6.9  -92.5 +/- 6.6 |
| 10. |  | **D950N**  E156G  F157-  G142D  L452R  P681R  R158-  **T19R**  T478K  D614G | 84.45%  32.51%  32.51%  42.93%  97.17%  97.44%  32.51%  94.96%  97.17%  99.82% | 99.49%  98.87%  98.89%  62.02%  99.75%  100.00%  98.87%  99.47%  99.75%  100.00% | Regdanvimab | -210.546  -129.374  -109.4  -165.517  -119.704  -130.159  -82.0766  -129.804  -207.235  -109.15 | **-127.9 +/- 17.4**  -138.7 +/- 4.1  -123.6 +/- 7.0  -118.4 +/- 9.4  -121.2 +/- 6.7  -132.2 +/- 8.2  -130.0 +/- 1.9  **-117.8 +/- 10.6**  -130.4 +/- 15.3  -140.3 +/- 3.1 |
| 11. |  | D950N  E156G  F157-  **G142D**  L452R  **P681R**  R158-  T19R  T478K  D614G | 84.45%  32.51%  32.51%  42.93%  97.17%  97.44%  32.51%  94.96%  97.17%  99.82% | 99.49%  98.87%  98.89%  62.02%  99.75%  100.00%  98.87%  99.47%  99.75%  100.00% | Tixagevimab | -179.155  -117.859  -178.119  -146.971  -93.0666  -92.8114  -186.404  -52.2907  -122.397  -6.46754 | -42.2 +/- 4.4  -49.8 +/- 17.8  -89.1 +/- 4.7  **-22.6 +/- 10.3**  -78.9 +/- 0.7  **-90.4 +/- 4.0**  -31.6 +/- 3.8  -24.2 +/- 11.3  -89.6 +/- 9.9  -72.1 +/- 5.6 |
| 12. |  | D950N  **E156G**  F157-  G142D  L452R  P681R  R158-  T19R  T478K  **D614G** | 84.45%  32.51%  32.51%  42.93%  97.17%  97.44%  32.51%  94.96%  97.17%  99.82% | 99.49%  98.87%  98.89%  62.02%  99.75%  100.00%  98.87%  99.47%  99.75%  100.00% | Cilgavimab | -198.851  -229.383  -221.675  -270.639  -60.8845  -97.7285  -240.347  -0.736647  -61.9131  -148.188 | -108.1 +/- 5.8  **-112.2 +/- 11.4**  -104.6 +/- 9.4  -112.0 +/- 14.4  -80.1 +/- 5.1  -76.2 +/- 5.3  -99.5 +/- 8.1  -77.3 +/- 7.2  -67.3 +/- 4.2  **-66.1 +/- 17.2** |
| 13. |  | D950N  E156G  F157-  G142D  L452R  P681R  **R158-**  T19R  T478K  **D614G** | 84.45%  32.51%  32.51%  42.93%  97.17%  97.44%  32.51%  94.96%  97.17%  99.82% | 99.49%  98.87%  98.89%  62.02%  99.75%  100.00%  98.87%  99.47%  99.75%  100.00% | Etesevimab | -164.67  -146.671  -99.3193  -178.584  -86.2803  -109.011  -191.42  -57.9951  -94.635  -113.256 | -55.2 +/- 9.1  -66.8 +/- 6.1  -107.0 +/- 8.2  -48.8 +/- 2.6  -109.4 +/- 2.2  -110.7 +/- 0.4  **-38.9 +/- 18.9**  -57.5 +/- 5.7  -110.7 +/- 3.5  **-139.0 +/- 3.2** |
| 14. |  | D950N  E156G  F157-  **G142D**  L452R  P681R  R158-  T19R  **T478K**  D614G | 84.45%  32.51%  32.51%  42.93%  97.17%  97.44%  32.51%  94.96%  97.17%  99.82% | 99.49%  98.87%  98.89%  62.02%  99.75%  100.00%  98.87%  99.47%  99.75%  100.00% | Casirivimab | -133.456  -132.076  -13.8008  -221.763  -6.43515  -39.9829  -29.3931  -8.42325  -147.837  -20.9452 | -38.2 +/- 4.7  -58.8 +/- 12.5  -69.3 +/- 3.7  **-37.3 +/- 9.3**  -62.5 +/- 3.8  -67.2 +/- 1.7  -44.9 +/- 11.3  -40.7 +/- 7.9  **-74.9 +/- 5.0**  -59.7 +/- 4.4 |
| 15. |  | D950N  E156G  F157-  G142D  L452R  **P681R**  R158-  **T19R**  T478K  D614G | 84.45%  32.51%  32.51%  42.93%  97.17%  97.44%  32.51%  94.96%  97.17%  99.82% | 99.49%  98.87%  98.89%  62.02%  99.75%  100.00%  98.87%  99.47%  99.75%  100.00% | Imdevimab | -222.453  -186.126  -78.8986  -180.264  -28.8199  -70.1897  -194.369  -178.299  -0.773568  -3.93831 | -48.1 +/- 15.0  -52.0 +/- 8.6  -79.3 +/- 8.5  -56.4 +/- 12.0  -78.6 +/- 3.5  **-82.6 +/- 5.3**  -43.6 +/- 21.6  **-37.7 +/- 16.9**  -79.8 +/- 3.9  -65.4 +/- 5.0 |
| 16. |  | D950N  E156G  F157-  G142D  L452R  P681R  **R158-**  T19R  T478K  **D614G** | 84.45%  32.51%  32.51%  42.93%  97.17%  97.44%  32.51%  94.96%  97.17%  99.82% | 99.49%  98.87%  98.89%  62.02%  99.75%  100.00%  98.87%  99.47%  99.75%  100.00% | Sotrovimab | -184.215  -221.401  -135.855  -201.898  -128.953  -141.872  -167.15  -149.639  -112.841  -117.134 | -128.9 +/- 3.6  -125.9 +/- 11.7  -127.2 +/- 10.7  -118.8 +/- 6.9  -127.6 +/- 8.6  -122.9 +/- 5.9  **-132.0 +/- 7.9**  -125.5 +/- 1.5  -122.9 +/- 5.7  **-67.1 +/- 14.4** |

^$$^Scores marked with bold and >100 are considered as strong binding/interaction while scores with values <100 are considered as weak binding.

**Table S2:** Biomolecular interactions amongst the spike proteins and low affinity monoclonal antibodies.

| **Interaction details** | | | | | | | | | | | |
| --- | --- | --- | --- | --- | --- | --- | --- | --- | --- | --- | --- |
| **Antigen residues** | **Fab residues** | **Matched with SAbPred predicted CDR residues** | **Distance (Å)** | **Antigen residues** | **Fab residues** | **Matched with SAbPred predicted CDR residues** | **Distance (Å)** | **Antigen residues** | **Fab residues** | **Matched with SAbPred predicted CDR residues** | **Distance (Å)** |
| **B.1.1.7 (Alpha)-Lineage of SARS-CoV-2 Strain** | | | | | | | | | | | |
| **T716I-cilgavimab** | | | | **S982A-tixagevimab** | | | | **S982A-etsevimab** | | | |
| **Hydrogen Bond** | | | | | | | | | | | |
| LYS720 | ASP106 | Y | 1.49651 | GLU1399 | ARG87 |  | 1.89151 | THR589 | TYR32 |  | 1.86791 |
| GLN2563 | ASP106 | Y | 2.4356 | GLU1399 | ARG87 |  | 1.65791 | ASN593 | SER52 | Y | 1.99583 |
| ASP2912 | SER159 |  | 1.79356 | ASP1948 | ARG217 |  | 1.53988 | ARG834 | GLN145 | Y | 2.89492 |
| SER3186 | LYS162 |  | 1.77173 | LYS1396 | GLU66 |  | 1.63999 | ASP817 | SER146 | Y | 2.62657 |
| GLU2910 | TYR164 |  | 2.50612 | ASN1721 | TYR60 |  | 2.17716 | ASP817 | SER148 | Y | 1.90938 |
| SER3178 | ARG186 |  | 1.82212 | SER2057 | TYR154 | Y | 2.32109 | GLN936 | ARG149 | Y | 2.5033 |
| ASP3185 | GLY196 |  | 2.31355 | SER2057 | TYR154 | Y | 2.55723 | LYS812 | ARG149 | Y | 2.27975 |
| SER3189 | GLY198 |  | 2.07148 | ASN1942 | SER215 | Y | 2.63449 | SER926 | LEU172 | Y | 2.18944 |
| THR2927 | THR226 |  | 3.05112 | ASP1948 | ARG217 | Y | 2.93375 | ASN590 | ASN31 | Y | 2.98256 |
| LYS2559 | SER158 |  | 1.73781 | LYS1396 | GLU66 |  | 1.8811 | ARG834 | GLN145 | Y | 1.9428 |
| ASN2952 | ASP31 | Y | 2.18929 | ASN1721 | ILE70 |  | 2.02767 | ALA929 | TYR167 |  | 3.00212 |
| SER2953 | ASP31 | Y | 1.59174 | PRO1930 | SER189 |  | 3.644 | THR591 | SER30 | Y | 3.40409 |
| SER2953 | ASP31 | Y | 1.78712 |  |  |  |  | LYS812 | SER171 | Y | 3.26154 |
| THR2972 | VAL107 |  | 2.09124 |  |  |  |  | SER927 | LEU172 | Y | 3.64296 |
| LYS3182 | SER184 |  | 1.69034 |  |  |  |  |  |  |  |  |
| THR3190 | ASN161 |  | 2.95181 |  |  |  |  |  |  |  |  |
| SER3192 | ASN161 |  | 1.80698 |  |  |  |  |  |  |  |  |
| SER3189 | GLY198 |  | 3.2909 |  |  |  |  |  |  |  |  |
| HIS3313 | VAL107 |  | 3.34782 |  |  |  |  |  |  |  |  |
| **Electrostatic Bond** | | | | | | | | | | | |
|  |  |  |  | GLU1399 | ARG67 |  | 5.52524 |  |  |  |  |
|  |  |  |  | GLU1399 | ARG87 |  | 3.88204 |  |  |  |  |
| **Hydrophobic Bond (Amide- π)** | | | | | | | | | | | |
| LYS773; GLN774 | TYR105 | Y | 4.72529 |  |  |  |  |  |  |  |  |
| **Hydrophobic Bond (Alkyl)** | | | | | | | | | | | |
| LYS3182 | ARG186 |  | 5.22671 |  |  |  |  |  |  |  |  |
| ALA1002 | PRO108 | Y | 5.34276 |  |  |  |  |  |  |  |  |
| ALA1003 | PRO108 | Y | 4.66189 |  |  |  |  |  |  |  |  |
| ARG1006 | PRO108 | Y | 4.94885 |  |  |  |  |  |  |  |  |
| ALA1013 | LEU109 | Y | 4.26295 |  |  |  |  |  |  |  |  |
| **Hydrophobic Bond (π-Alkyl)** | | | | | | | | | | | |
| LYS773 | TYR105 | Y | 4.43827 |  |  |  |  | ALA929 | TYR167 |  | 4.92471 |
|  | | | | | | | | | | | |
| **B.1.617.2 (Delta)-Lineage of SARS-CoV-2 Strain** | | | | | | | | | | | |
| **T19R-bamlanivimab** | | | | **G142D-tixagevimab** | | | | **T19R-regdanivimab** | | | |
| **Hydrogen Bond** | | | | | | | | | | | |
| ASP274 | LYS74 |  | 2.99801 | ASP2054 | ARG176 | Y | 1.97454 | GLN3259 | TYR105 | Y | 1.97956 |
| ASN590 | SER77 |  | 2.87171 | ASN1724 | SER55 | Y | 2.46502 | GLN3198 | TYR107 | Y | 2.3028 |
| ASN940 | HIS104 | Y | 2.49505 | THR1720 | ASN57 | Y | 2.46351 | ASP2536 | ARG177 | Y | 2.44025 |
| ASN811 | TYR171 | Y | 1.85278 | ASN1724 | ASN57 | Y | 2.14378 | SER2295 | SER179 | Y | 2.26277 |
| SER926 | SER178 | Y | 2.4646 | ASN1398 | GLN65 |  | 2.99786 | ASN2852 | LYS189 |  | 1.81936 |
| SER926 | GLY179 |  | 2.83302 | SER1928 | ARG139 |  | 2.53783 | SER3189 | SER216 | Y | 1.78038 |
| GLN791 | SER182 |  | 1.96005 | SER1928 | ARG139 |  | 2.13018 | GLN749 | TYR105 | Y | 2.84364 |
| LYS265 | LEU55 | Y | 2.64143 | ASP1961 | GLN148 | Y | 1.76024 | ARG752 | TYR105 | Y | 2.17228 |
| ASN590 | SER77 |  | 1.98888 | ALA1947 | SER149 | Y | 186623 | ARG752 | TYR105 | Y | 2.73983 |
| LYS812 | SER178 | Y | 1.89097 | ASP1948 | SER151 | Y | 1.69309 | PHE2469 | ASP183 |  | 2.55393 |
| ASN940 | GLU102 | Y | 2.2882 | ASN1942 | SER152 | Y | 2.89616 | LYS2527 | ARG177 | Y | 1.79996 |
| ASN590 | LYS74 |  | 3.31209 | SER2058 | SER175 | Y | 2.6493 | THR2853 | LYS189 |  | 2.39694 |
| LYS822 | TYR105 | Y | 3.32188 | SER2057 | ARG176 | Y | 1.92213 | ALA3191 | ASN153 | Y | 1.91717 |
|  |  |  |  | ASN1942 | GLY188 |  | 1.70286 | SER3188 | SER216 | Y | 3.13567 |
|  |  |  |  | ASN1398 | ARG217 | Y | 1.84777 |  |  |  |  |
|  |  |  |  | ASN1398 | ARG217 | Y | 2.39799 |  |  |  |  |
|  |  |  |  | LYS1396 | ASN59 |  | 2.82274 |  |  |  |  |
|  |  |  |  | ASN1398 | GLN65 |  | 1.86829 |  |  |  |  |
|  |  |  |  | GLU1399 | GLN62 |  | 2.48912 |  |  |  |  |
|  |  |  |  | ASN1400 | GLU66 |  | 2.77182 |  |  |  |  |
|  |  |  |  | ASN1724 | ASN57 | Y | 2.75491 |  |  |  |  |
|  |  |  |  | SER2057 | SER174 | Y | 1.89606 |  |  |  |  |
| **Electrostatic Bond** | | | | | | | | | | | |
|  |  |  |  | ASP1926 | ARG139 |  | 5.37096 | ASP3092 | ARG99 |  | 5.00651 |
|  |  |  |  | GLU1399 | ARG217 |  | 4.58657 | GLU2558 | LYS176 | Y | 5.3582 |
|  |  |  |  |  |  |  |  | ASP2536 | ARG177 | Y | 4.79734 |
|  |  |  |  |  |  |  |  | ASP2536 | ARG177 | Y | 2.67948 |
|  |  |  |  |  |  |  |  | PHE2555 | ARG177 | Y | 4.34243 |
| **Hydrophobic Bond (π- π)** | | | | | | | | | | | |
|  |  |  |  |  |  |  |  | PHE3082 | TRP55 | Y | 4.76975 |
|  |  |  |  |  |  |  |  | PHE3082 | TRP55 | Y | 4.93226 |
| **Hydrophobic Bond (π-Alkyl)** | | | | | | | | | | | |
| ILE837 | TYR105 | Y | 4.35303 |  |  |  |  | ALA3078 | TRP55 | Y | 4.91252 |
|  |  |  |  |  |  |  |  | ILE3099 | PHE103 | Y | 5.49833 |
|  |  |  |  |  |  |  |  | ALA753 | TYR105 | Y | 5.37745 |
|  |  |  |  |  |  |  |  | LEU999 | TYR105 | Y | 5.39796 |

**Table S3:List of chimeric monoclonal antibodies conceived by using different mAbs**

| **Sl.No** | **Chimeric Antibody** | **mAb Framework** | **mAb CDR** | **CDR Sequence** |
| --- | --- | --- | --- | --- |
|  | Bamlanivimab-framework-Cilgavimab-CDRH3 | Bamlanivimab | Cilgavimab | TTAGSYYYDTVGPGLPEGKFDY |
|  | Bamlanivimab-framework-Regdanivimab-CDRH3 | Bamlanivimab | Regdanivimab | ARIPGFLRYRNRYYYYGMDV |
|  | Bamlanivimab-framework-Sotrovimab-CDRH3 | Bamlanivimab | Sotrovimab | ARDYTRGAWFGESLIGGFDN |
|  | Bamlanivimab-framework-Tixagevimab-CDRH3 | Bamlanivimab | Tixagevimab | AAPYCSSISCNDGFDI |
|  | Cilgavimab-framework-Bamlanivimab-CDRH3 | Cilgavimab | Bamlanivimab | ARGYYEARHYYYYYAMDV |
|  | Cilgavimab-framework-Regdanivimab-CDRH3 | Cilgavimab | Regdanivimab | ARIPGFLRYRNRYYYYGMDV |
|  | Cilgavimab-framework-Sotrovimab-CDRH3 | Cilgavimab | Sotrovimab | ARDYTRGAWFGESLIGGFDN |
|  | Cilgavimab-framework-Tixagevimab-CDRH3 | Cilgavimab | Tixagevimab | AAPYCSSISCNDGFDI |
|  | Regdanivimab-framework-Bamlanivimab-CDRH3 | Regdanivimab | Bamlanivimab | ARGYYEARHYYYYYAMDV |
|  | Regdanivimab-framework-Cilgavimab-CDRH3 | Regdanivimab | Cilgavimab | TTAGSYYYDTVGPGLPEGKFDY |
|  | Regdanivimab-framework-Sotrovimab-CDRH3 | Regdanivimab | Sotrovimab | ARDYTRGAWFGESLIGGFDN |
|  | Regdanivimab-framework-Tixagevimab-CDRH3 | Regdanivimab | Tixagevimab | AAPYCSSISCNDGFDI |
|  | Sotrovimab-framework-Bamlanivimab-CDRH3 | Sotrovimab | Bamlanivimab | ARGYYEARHYYYYYAMDV |
|  | Sotrovimab-framework-Cilgavimab-CDRH3 | Sotrovimab | Cilgavimab | TTAGSYYYDTVGPGLPEGKFDY |
|  | Sotrovimab-framework-Regdanivimab-CDRH3 | Sotrovimab | Regdanivimab | ARIPGFLRYRNRYYYYGMDV |
|  | Sotrovimab-framework-Tixagevimab-CDRH3 | Sotrovimab | Tixagevimab | AAPYCSSISCNDGFDI |
|  | Tixagevimab-framework-Bamlanivimab-CDRH3 | Tixagevimab | Bamlanivimab | ARGYYEARHYYYYYAMDV |
|  | Tixagevimab-framework-Cilgavimab-CDRH3 | Tixagevimab | Cilgavimab | TTAGSYYYDTVGPGLPEGKFDY |
|  | Tixagevimab-framework-Regdanivimab-CDRH3 | Tixagevimab | Regdanivimab | ARIPGFLRYRNRYYYYGMDV |
|  | Tixagevimab-framework-Sotrovimab-CDRH3 | Tixagevimab | Sotrovimab | ARDYTRGAWFGESLIGGFDN |

**Table S4A: List of all the developed chimeric mAbs and their level of efficacy against B.1.1.7 and B.1.617.2 lineages.**

| **Sl. No.** | **Lineage of SARS-CoV-2 Strain** | **Spike protein with mutation** | **Interacting Chimeric Monoclonal Antibody** | **Binding affinity ΔG (kcal/mol)** | **Haddock 2.4 score** |
| --- | --- | --- | --- | --- | --- |
|  |  |  |  |  |  |
| 1. | B.1.1.7  (Alpha) | A570D  D614G  D1118H  H69-  N501Y  P681H  S982A  T716I  V70-  Y144- | Bamlanivimab-framework-Cilgavimab-CDRH3 | -14.7  -14.6  -14.1  -15.0  -15.7  -13.5  -15.5  -13.8  -14.2  -15.2 | -44.0 +/- 22.9  -104.9 +/- 16.3  -65.9 +/- 6.7  -106.1 +/- 4.8  -81.5 +/- 41.1  -90.2 +/- 5.9  -88.7 +/- 21.6  -73.9 +/- 8.0  -96.6 +/- 3.1  -91.5 +/- 21.0 |
| 2. |  | A570D  D614G  D1118H  H69-  N501Y  P681H  S982A  T716I  V70-  Y144- | Bamlanivimab-framework-Regdanivimab-CDRH3 | -14.8  -12.3  -13.1  -10.2  -12.6  -13.2  -13.4  -11.5  -11.1  -14.0 | -124.5 +/- 12.7  -101.0 +/- 5.7  -111.4 +/- 2.7  -107.0 +/- 5.0  -110.4 +/- 5.3  -110.4 +/- 5.2  -103.1 +/- 7.6  -99.8 +/- 6.4  -100.5 +/- 5.1  -117.1 +/- 14.5 |
| 3. |  | A570D  D614G  D1118H  H69-  N501Y  P681H  S982A  T716I  V70-  Y144- | Bamlanivimab-framework-Sotrovimab-CDRH3 | -11.1  -12.0  -12.5  -13.2  -12.3  -14.3  -13.0  -14.2  -14.2  -12.0 | -108.1 +/- 1.6  -115.5 +/- 4.2  -119.2 +/- 3.0  -115.4 +/- 4.8  -120.2 +/- 5.8  -105.3 +/- 7.7  -114.3 +/- 2.8  -118.8 +/- 7.9  -108.0 +/- 7.4  -115.5 +/- 4.2 |
| 4. |  | A570D  D614G  D1118H  H69-  N501Y  P681H  S982A  T716I  V70-  Y144- | Bamlanivimab-framework-Tixagevimab-CDRH3 | -10.7  -12.5  -10.5  -12.0  -10.9  -13.6  -10.7  -10.1  -11.5  -9.2 | -41.0 +/- 2.4  -79.7 +/- 6.8  -48.0 +/- 3.8  -53.6 +/- 9.2  -34.0 +/- 11.8  -59.9 +/- 12.0  -52.8 +/- 7.4  -36.8 +/- 1.5  -55.9 +/- 8.7  -42.2 +/- 8.7 |
| 5. |  | A570D  D614G  D1118H  H69-  N501Y  P681H  S982A  T716I  V70-  Y144- | Cilgavimab-framework-Bamlanivimab-CDRH3 | -13.4  -11.3  -12.7  -13.2  -12.3  -12.7  -14.3  -12.0  -12.2  -10.0 | -106.3 +/- 9.7  -112.8 +/- 11.9  -96.0 +/- 9.6  -129.4 +/- 4.8  -90.6 +/- 15.3  -124.9 +/- 5.9  -112.2 +/- 5.6  -98.2 +/- 9.1  -115.2 +/- 6.2  -80.1 +/- 4.9 |
| 6. |  | A570D  D614G  D1118H  H69-  N501Y  P681H  S982A  T716I  V70-  Y144- | Cilgavimab-framework-Regdanivimab-CDRH3 | -14.6  -12.7  -11.1  -14.0  -10.0  -14.0  -9.7  -11.5  -14.0  -11.2 | -123.6 +/- 3.2  -87.1 +/- 18.6  -127.5 +/- 9.2  -122.7 +/- 6.1  -124.6 +/- 8.5  -120.2 +/- 3.4  -115.5 +/- 8.2  -94.7 +/- 3.5  -114.5 +/- 3.9  -95.4 +/- 3.5 |
| 7. |  | A570D  D614G  D1118H  H69-  N501Y  P681H  S982A  T716I  V70-  Y144- | Cilgavimab-framework-Sotrovimab-CDRH3 | -11.7  -11.6  -11.9  -14.4  -12.9  -13.9  -11.9  -8.2  -13.1  -11.9 | -89.2 +/- 5.4  -92.3 +/- 16.4  -71.8 +/- 4.3  -113.2 +/- 11.0  -89.4 +/- 14.5  -122.9 +/- 12.4  -100.8 +/- 4.7  -80.6 +/- 0.6  -95.3 +/- 13.4  -76.5 +/- 14.1 |
| 8. |  | A570D  D614G  D1118H  H69-  N501Y  P681H  S982A  T716I  V70-  Y144- | Cilgavimab-framework-Tixagevimab-CDRH3 | -11.6  -10.7  -11.0  -10.0  -13.8  -8.2  -11.3  -10.1  -10.0  -10.9 | -76.6 +/- 2.4  -92.1 +/- 9.5  -72.4 +/- 2.2  -84.3 +/- 3.3  -77.5 +/- 6.3  -81.1 +/- 1.3  -70.9 +/- 5.5  -91.7 +/- 4.2  -81.4 +/- 1.6  -87.5 +/- 5.6 |
| 9. |  | A570D  D614G  D1118H  H69-  N501Y  P681H  S982A  T716I  V70-  Y144- | Regdanivimab-framework-Bamlanivimab-CDRH3 | -12.2  -12.0  -9.9  -11.9  -10.7  -14.0  -12.7  -11.3  -10.7  -10.5 | -118.4 +/- 11.7  -107.0 +/- 15.5  -109.6 +/- 13.0  -103.3 +/- 8.4  -108.5 +/- 11.9  -118.9 +/- 14.6  -104.4 +/- 21.0  -106.1 +/- 7.6  -102.7 +/- 4.6  -116.7 +/- 13.1 |
| 10. |  | A570D  D614G  D1118H  H69-  N501Y  P681H  S982A  T716I  V70-  Y144- | Regdanivimab-framework-Cilgavimab-CDRH3 | -12.2  -12.6  -12.1  -13.9  -11.7  -12.4  -12.5  -11.8  -12.7  -14.5 | -42.6 +/- 4.1  -97.7 +/- 3.0  -41.0 +/- 8.1  -111.0 +/- 2.4  -33.4 +/- 12.2  -104.6 +/- 4.6  -30.6 +/- 23.0  -30.6 +/- 8.0  -103.1 +/- 4.1  -39.6 +/- 8.2 |
| 11 |  | A570D  D614G  D1118H  H69-  N501Y  P681H  S982A  T716I  V70-  Y144- | Regdanivimab-framework-Sotrovimab-CDRH3 | -14.4  -12.2  -13.5  -13.3  -11.0  -12.8  -13.2  -12.7  -11.9  -11.6 | -122.1 +/- 2.6  -114.5 +/- 3.3  -113.1 +/- 16.7  -121.0 +/- 4.1  -118 +/- 9.1  -110 +/- 3.4  -123.0 +/- 12.9  -89.5 +/- 26.6  -120.0 +/- 5.5  -120.5 +/- 3.6 |
| 12. |  | A570D  D614G  D1118H  H69-  N501Y  P681H  S982A  T716I  V70-  Y144- | Regdanivimab-framework-Tixagevimab-CDRH3 | -12.4  -14.1  -14.2  -11.8  -12.6  -12.8  -10.8  -11.3  -14.1  -11.7 | -48.6 +/- 12.4  -116.5 +/- 3.3  -87.1 +/- 8.6  -73.5 +/- 7.9  -37.0 +/- 25.5  -62.6 +/- 4.5  -22.7 +/- 0.8  -45.3 +/- 13.5  -79.2 +/- 9.4  -47.9 +/- 8.5 |
| 13. |  | A570D  D614G  D1118H  H69-  N501Y  P681H  S982A  T716I  V70-  Y144- | Sotrovimab-framework-Bamlanivimab-CDRH3 | -13.0  -12.1  -13.5  -10.3  -9.2  -13.5  -14.3  -11.7  -11.7  -13.7 | -104.2 +/- 20.0  -102.3 +/- 14.0  -97.8 +/- 13.5  -84.9 +/- 12.9  -35.8 +/- 5.9  -118.8 +/- 16.3  -97.7 +/- 9.8  -82.3 +/- 47.0  -104.1 +/- 7.6  -74.9 +/- 11.2 |
| 14. |  | A570D  D614G  D1118H  H69-  N501Y  P681H  S982A  T716I  V70-  Y144- | Sotrovimab-framework-Cilgavimab-CDRH3 | -12.5  -13.4  -14.5  -12.3  -10.0  -14.3  -18.2  -8.7  -12.5  -13.9 | -33.1 +/- 3.5  -58.3 +/- 13.9  -15.1 +/- 20.3  -58.1 +/- 5.3  -2.3 +/- 6.7  -59.5 +/- 8.6  -38.7 +/- 43.4  16.8 +/- 2.7  -47.1 +/- 7.6  -56.0 +/- 8.1 |
| 15. |  | A570D  D614G  D1118H  H69-  N501Y  P681H  S982A  T716I  V70-  Y144- | Sotrovimab-framework-Regdanivimab-CDRH3 | -12.0  -12.2  -13.4  -11.5  -12.3  -11.4  -12.8  -12.0  -11.9  -12.6 | -105.1 +/- 4.9  -114.5 +/- 3.3  -111.6 +/- 8.6  -108.0 +/- 9.4  -114.7 +/- 3.0  -99.6 +/- 10.0  -108.3 +/- 9.0  -105.7 +/- 8.9  -107.9 +/- 12.4  -109.4 +/- 14.0 |
| 16. |  | A570D  D614G  D1118H  H69-  N501Y  P681H  S982A  T716I  V70-  Y144- | Sotrovimab-framework-Tixagevimab-CDRH3 | -11.5  -10.6  -13.9  -13.2  -10.2  -9.4  -11.2  -13.4  -14.7  -7.9 | -16.3 +/- 27.1  -97.1 +/- 8.1  -21.6 +/- 15.3  -84.0 +/- 15.3  -16.3 +/- 8.4  -85.5 +/- 2.0  -24.2 +/- 11.4  -19.4 +/- 12.3  -76.0 +/- 9.5  -18.4 +/- 7.4 |
| 17. |  | A570D  D614G  D1118H  H69-  N501Y  P681H  S982A  T716I  V70-  Y144- | Tixagevimab-framework-Bamlanivimab-CDRH3 | -13.2  -13.9  -11.5  -13.0  -12.9  -13.4  -11.5  -12.9  -12.4  -13.0 | -102.5 +/- 7.3  -105.0 +/- 5.3  -106.2 +/- 12.4  -126.6 +/- 5.6  -114.4 +/- 8.9  -123.0 +/-2.2  -125.2 +/- 26.9  -104.6 +/- 10.1  -126.2 +/- 7.0  -105.3 +/- 6.2 |
| 18. |  | A570D  D614G  D1118H  H69-  N501Y  P681H  S982A  T716I  V70-  Y144- | Tixagevimab-framework-Cilgavimab-CDRH3 | -11.3  -12.9  -12.3  -15.0  -11.5  -14.2  -11.6  -10.8  -8.1  -10.9 | -57.1 +/- 10.5  -104.8 +/- 2.8  -45.8 +/- 10.3  -62.0 +/- 3.9  -25.9 +/- 9.7  -64.6 +/- 6.4  -30.9 +/- 13.0  -38.6 +/- 4.2  -57.5 +/- 6.6  -61.8 +/- 2.6 |
| 19. |  | A570D  D614G  D1118H  H69-  N501Y  P681H  S982A  T716I  V70-  Y144- | Tixagevimab-framework-Regdanivimab-CDRH3 | -11.1  -9.1  -13.6  -9.6  -11.1  -10.1  -11.5  -12.5  -9.2  -11.2 | -90.7 +/- 6.7  -96.5 +/- 1.6  -96.7 +/- 4.9  -104.2 +/- 1.7  -70.8 +/- 7.4  -107.7 +/- 3.7  -101.0 +/- 8.8  -86.0 +/- 4.4  -105.8 +/- 3.1  -87.0 +/- 6.6 |
| 20. |  | A570D  D614G  D1118H  H69-  N501Y  P681H  S982A  T716I  V70-  Y144- | Tixagevimab-framework-Sotrovimab-CDRH3 | -10.5  -12.5  -11.5  -13.8  -12.6  -13.0  -11.7  -13.2  -12.8  -13.2 | -41.8 +/- 12.1  -99.3 +/- 4.5  -56.8 +/- 7.4  -67.8 +/- 10.2  83.5 +/- 8.8  82.5 +/- 7.6  -82.7 +/- 2.7  -75.6 +/- 10.6  -96.7 +/- 18.8  -82.4 +/- 6.0 |
| 21. | B.1.617.2  (Delta) | D950N  E156G  F157-  G142D  L452R  P681R  R158-  T19R  T478K  D614G | Bamlanivimab-framework-Cilgavimab-CDRH3 | -11.9  -11.8  -13.0  -12.2  -12.8  -12.6  -12.7  -11.2  -13.5  -14.6 | -68.5 +/- 18.8  -71.5 +/- 4.6  -105.5 +/- 4.4  -73.4 +/- 14.9  -111.2 +/- 10.1  -108.7 +/- 5.2  -69.7 +/- 10.2  -36.7 +/- 5.8  -109.7 +/- 5.1  -104.9 +/- 16.3 |
| 22. |  | D950N  E156G  F157-  G142D  L452R  P681R  R158-  T19R  T478K  D614G | Bamlanivimab-framework-Regdavimab-CDRH3 | -12.1  -14.1  -13.0  -12.6  -12.2  -10.5  -14.7  -14.6  -10.1  -12.3 | -120.8 +/- 6.9  -116.2 +/- 13.3  -102.1 +/- 5.4  -111.8 +/- 6.1  -92.7 +/- 5.4  -100.8 +/- 2.8  -122.1 +/- 11.0  -99.6 +/- 11.1  -102.2 +/- 7.8  -101.0 +/- 5.7 |
| 23. |  | D950N  E156G  F157-  G142D  L452R  P681R  R158-  T19R  T478K  D614G | Bamlanivimab-framework-Sotrovimab-CDRH3 | -13.4  -11.4  -11.5  -11.5  -12.6  -13.2  -13.1  -13.9  -12.7  -12.0 | -116.2 +/- 1.3  -113.6 +/- 6.4  -77.2 +/- 10.7  -100.7 +/- 4.6  -99.3 +/- 4.2  -106.9 +/- 5.5  -98.9 +/- 4.8  -113.4 +/- 10.7  -119.4 +/- 11.3  -115.5 +/- 4.2 |
| 24. |  | D950N  E156G  F157-  G142D  L452R  P681R  R158-  T19R  T478K  D614G | Bamlanivimab-framework-Tixagevimab-CDRH3 | -9.8  -10.4  -14.3  -10.0  -11.8  -12.4  -11.6  -11.5  -13.7  -12.5 | -47.4 +/- 12.7  -28.1 +/- 8.9  -66.2 +/- 7.5  -39.4 +/- 6.9  -57.0 +/- 1.2  -55.1 +/- 2.2  -38.9 +/- 16.6  -20.6 +/- 2.8  -63.6 +/-8.2  -79.7 +/- 6.8 |
| 25. |  | D950N  E156G  F157-  G142D  L452R  P681R  R158-  T19R  T478K  D614G | Cilgavimab-framework-Bamlanivimab-CDRH3 | -11.6  -11.5  -12.7  -11.9  -12.6  -12.6  -12.9  -14.3  -13.2  -11.3 | -85.7 +/- 9.0  -92.1 +/- 5.5  -104.1 +/- 4.2  -83.7 +/- 4.9  -103.6 +/- 12.6  -124.4 +/- 2.9  -97.4 +/- 20.0  -99.9 +/- 7.4  -124.5 +/- 5.1  -112.8 +/- 11.9 |
| 26. |  | D950N  E156G  F157-  G142D  L452R  P681R  R158-  T19R  T478K  D614G | Cilgavimab-framework-Regdanivimab-CDRH3 | -11.1  -13.1  -14.3  -10.6  -12.1  -13.9  -9.6  -9.3  -12.2  -12.7 | -116.5 +/- 5.0  -117.4 +/- 7.4  -112.6 +/- 14.3  -123.8 +/- 14.1  -114.6 +/- 1.4  -129.8 +/- 8.4  -102.3 +/- 14.2  -105.4 +/- 4.1  -109.8 +/- 10.1  -87.1 +/- 18.6 |
| 27. |  | D950N  E156G  F157-  G142D  L452R  P681R  R158-  T19R  T478K  D614G | Cilgavimab-framework-Sotrovimab-CDRH3 | -11.3  -10.2  -12.6  -10.7  -17.5  -12.6  -12.9  -12.6  -12.2  -11.6 | -88.3 +/- 5.8  -87.1 +/- 2.1  -115.8 +/- 6.4  -85.6 +/- 4.6  -117.1 +/- 8.3  -116.6 +/- 3.7  -91.2 +/- 9.1  -81.9 +/- 3.3  -109.7 +/- 9.4  -92.3 +/- 16.4 |
| 28. |  | D950N  E156G  F157-  G142D  L452R  P681R  R158-  T19R  T478K  D614G | Cilgavimab-framework-Tixagevimab-CDRH3 | -11.9  -11.7  -11.0  -13.3  -8.7  -8.8  -12.9  -11.9  -13.1  -10.7 | -82.6 +/- 3.3  -89.6 +/- 10.5  -86.7 +/- 6.4  -78.6 +/- 6.1  -82.7 +/- 2.5  -83.8 +/- 2.7  -97.5 +/- 5.7  -85.4 +/- 8.2  -79.7 +/- 16.8  -92.1 +/- 9.5 |
| 29. |  | D950N  E156G  F157-  G142D  L452R  P681R  R158-  T19R  T478K  D614G | Regdanivimab-framework-Bamlanivimab-CDRH3 | -11.8  -12.5  -13.1  -12.0  -12.0  -11.2  -11.4  -12.1  -12.4  -12.0 | -121.2 +/- 15.5  -124.9 +/- 4.2  -113.2 +/- 3.3  -109.9 +/- 29.4  -119.0 +/- 5.9  -109.9 +/- 19.3  -108.7 +/- 5.4  -119.8 +/- 2.5  -113.2 +/- 23.5  -107.0 +/- 15.5 |
| 30. |  | D950N  E156G  F157-  G142D  L452R  P681R  R158-  T19R  T478K  D614G | Regdanivimab-framework-Cilgavimab-CDRH3 | -12.0  -9.5  -11.2  -10.2  -12.9  -12.1  -13.1  -11.3  -12.5  -12.6 | -54.0 +/- 29.9  -53.6 +/- 21.5  -102.4 +/- 4.7  -42.9 +/- 7.0  -113.6 +/- 3.2  -125.0 +/- 2.8  -46.0 +/- 7.9  -32.8 +/- 7.4  -117.1 +/- 1.4  -97.7 +/- 3.0 |
| 31. |  | D950N  E156G  F157-  G142D  L452R  P681R  R158-  T19R  T478K  D614G | Regdanivimab-framework-Sotrovimab-CDRH3 | -13.2  -11.8  -13.8  -13.4  -12.1  -13.5  -12.2  -14.1  -11.5  -12.2 | -114.7 +/- 6.8  -95.2 +/- 5.5  -101.6 +/- 5.3  -117.2 +/- 6.5  -113.5 +/- 3.7  -122.1 +/- 4.6  -102.0 +/- 7.0  -120.5 +/- 11.4  -119.1 +/- 10.3  -114.5 +/- 3.3 |
| 32. |  | D950N  E156G  F157-  G142D  L452R  P681R  R158-  T19R  T478K  D614G | Regdanivimab-framework-Tixagevimab-CDRH3 | -8.9  -11.2  -12.2  -8.1  -12.2  -15.1  -8.9  -9.7  -13.9  -14.1 | -40.8 +/- 8.6  -35.7 +/- 7.3  -69.2 +/- 6.9  -41.7 +/- 15.7  -73.8 +/- 3.4  -81.1 +/- 9.3  -36.6 +/- 6.5  -40.5 +/- 11.5  -82.7 +/- 10.0  -116.5 +/- 3.3 |
| 33. |  | D950N  E156G  F157-  G142D  L452R  P681R  R158-  T19R  T478K  D614G | Sotrovimab-framework-Bamlanivimab-CDRH3 | -12.5  -12.1  -12.0  -12.2  -9.7  -8.5  -12.0  -12.3  -13.2  -12.1 | -92.0 +/- 18.8  -95.8 +/- 7.3  -101.9 +/- 7.7  -108.2 +/- 13.9  -99.4 +/- 12.1  -108.8 +/- 5.6  -91.8 +/- 12.4  -79.7 +/- 7.1  -101.0 +/- 22.4  -102.3 +/- 14.0 |
| 34. |  | D950N  E156G  F157-  G142D  L452R  P681R  R158-  T19R  T478K  D614G | Sotrovimab-framework-Cilgavimab-CDRH3 | -12.3  -11.8  -12.8  -13.3  -12.6  -11.7  -8.7  -12.3  -13.5  -13.4 | -30.1 +/- 24.0  -3.2 +/- 41.2  -44.2 +/- 10.9  -18.8 +/- 10.7  -68.7 +/- 5.7  -49.2 +/- 1.9  21.5 +/- 5.1  -22.6 +/- 13.3  -74.4 +/- 7.9  -58.3 +/- 13.9 |
| 35. |  | D950N  E156G  F157-  G142D  L452R  P681R  R158-  T19R  T478K  D614G | Sotrovimab-framework-Regdanivimab-CDRH3 | -11.5  -11.9  -12.1  -10.5  -10.9  -10.2  -12.7  -11.7  -12.8  -12.2 | -123.7 +/- 6.9  -107.9 +/- 6.0  -104.8 +/- 7.1  -117.2 +/- 5.3  -98.8 +/- 8.3  -104.3 +/- 2.0  -121.6 +/- 8.8  -117.6 +/- 14.3  -99.9 +/- 5.1  -114.5 +/- 3.3 |
| 36. |  | D950N  E156G  F157-  G142D  L452R  P681R  R158-  T19R  T478K  D614G | Sotrovimab-framework-Tixagevimab-CDRH3 | -12.2  -11.6  -11.0  -12.2  -10.2  -13.0  -10.7  -9.2  -8.4  -10.6 | -23.3 +/- 20.8  -18.3 +/- 25.2  85.5 +/- 5.5  -38.3 +/- 20.1  -81.4 +/- 3.6  -83.6 +/- 10.7  -23.9 +/- 5.0  -31.7 +/- 12.6  -84.3 +/- 5.8  -97.1 +/- 8.1 |
| 37. |  | D950N  E156G  F157-  G142D  L452R  P681R  R158-  T19R  T478K  D614G | Tixagevimab-framework-Bamlanivimab-CDRH3 | -12.0  -13.3  -12.5  -12.7  -12.0  -12.1  -11.1  -11.9  -12.2  -13.9 | -119.9 +/- 8.6  -112.6 +/- 5.0  -122.3 +/- 1.7  -108.9 +/- 12.0  -121.0 +/- 5.9  -116.3 +/- 4.7  -67.9 +/- 10.3  -119.0 +/- 8.2  -121.9 +/- 6.4  -105.0 +/- 5.3 |
| 38. |  | D950N  E156G  F157-  G142D  L452R  P681R  R158-  T19R  T478K  D614G | Tixagevimab-framework-Cilgavimab-CDRH3 | -12.8  -12.9  -11.6  -11.8  -10.7  -12.8  -10.4  -13.1  -10.5  -12.9 | -74.9 +/- 14.9  -43.7 +/- 12.8  -74.0 +/- 8.4  -82.6 +/- 27.8  62.3 +/- 7.4  -55.3 +/- 8.2  -26.0 +/- 3.3  -41.6 +/- 5.5  -52.8 +/- 2.8  -104.8 +/- 2.8 |
| 39. |  | D950N  E156G  F157-  G142D  L452R  P681R  R158-  T19R  T478K  D614G | Tixagevimab-framework-Regdanivimab-CDRH3 | -13.2  -13.3  -11.6  -13.8  -9.8  -10.9  -13.2  -12.5  -10.1  -9.1 | -71.9 +/- 11.0  -96.1 +/- 6.8  -101.8 +/- 3.6  -103.7 +/- 8.3  -108.7 +/- 3.5  -108.2 +/- 3.5  -110.1 +/- 2.1  -98.5 +/- 18.6  -110.2 +/- 6.7  -96.5 +/- 1.6 |
| 40. |  | D950N  E156G  F157-  G142D  L452R  P681R  R158-  T19R  T478K  D614G | Tixagevimab-framework-Sotrovimab-CDRH3 | -11.4  -12.0  -12.1  -10.0  -12.7  -14.7  -11.9  -9.5  -13.5  -12.5 | -81.1 +/- 4.4  -73.6 +/- 5.0  -76.1 +/- 9.8  -67.6 +/- 6.7  -73.5 +/- 4.0  -76.0 +/- 14.8  -82.5 +/- 4.6  -71.7 +/- 14.0  -76.4 +/- 5.2  -99.3 +/- 4.5 |

**Table S4B: List of all the developed chimeric mAbs and their level of efficacy against Delta plus variant (K417N).**

| **Sl. No.** | **Lineage of SARS-CoV-2 Strain** | **Spike protein with mutation** | **Interacting Chimeric Monoclonal Antibody** | **Binding affinity ΔG (kcal/mol)** | **Haddock 2.4 score** |
| --- | --- | --- | --- | --- | --- |
|  | B.1.617.2.1  (Delta plus) | K417N | Bamlanivimab-framework-Cilgavimab-CDRH3 | -12.5 | -103.8 +/- 2.7 |
|  |  |  | Bamlanivimab-framework-Regdavimab-CDRH3 | -12.2 | -102.5 +/- 5.4 |
|  |  |  | Bamlanivimab-framework-Sotrovimab-CDRH3 | -14.4 | 107.2 +/-6.3 |
|  |  |  | Bamlanivimab-framework-Tixagevimab-CDRH3 | -12.8 | -56.7 +/- 31.1 |
|  |  |  | Cilgavimab-framework-Bamlanivimab-CDRH3 | -12.8 | -106.0 +/- 6.2 |
|  |  |  | Cilgavimab-framework-Regdanivimab-CDRH3 | -13.1 | -103.4 +/- 4.7 |
|  |  |  | Cilgavimab-framework-Sotrovimab-CDRH3 | -11.1 | -88.5 +/- 5.8 |
|  |  |  | Cilgavimab-framework-Tixagevimab-CDRH3 | -9.1 | -82.2 +/- 2.1 |
|  |  |  | Regdanivimab-framework-Bamlanivimab-CDRH3 | -12.4 | -106.1 +/- 3.7 |
|  |  |  | Regdanivimab-framework-Cilgavimab-CDRH3 | -10.6 | -101.2 +/- 7.8 |
|  |  |  | Regdanivimab-framework-Sotrovimab-CDRH3 | 11.0 | -106.4 +/- 4.6 |
|  |  |  | Regdanivimab-framework-Tixagevimab-CDRH3 | -12.5 | -36.9 +/- 14.7 |
|  |  |  | Sotrovimab-framework-Bamlanivimab-CDRH3 | -10.7 | -99.5 +/- 13.2 |
|  |  |  | Sotrovimab-framework-Cilgavimab-CDRH3 | -10.4 | -74.8 +/- 29.2 |
|  |  |  | Sotrovimab-framework-Regdanivimab-CDRH3 | -11.5 | -108.8 +/- 1.2 |
|  |  |  | Sotrovimab-framework-Tixagevimab-CDRH3 | -11.2 | -23.9 +/- 4.7 |
|  |  |  | Tixagevimab-framework-Bamlanivimab-CDRH3 | -12.6 | -104.4 +/- 6.2 |
|  |  |  | Tixagevimab-framework-Cilgavimab-CDRH3 | -10.2 | -44.5 +/- 14.1 |
|  |  |  | Tixagevimab-framework-Regdanivimab-CDRH3 | -11.2 | -91.1 +/- 6.3 |
|  |  |  | Tixagevimab-framework-Sotrovimab-CDRH3 | -11.4 | -73.7 +/- 7.9 |

**Table S5:** Comparative SARS-CoV-2 neutralizing efficacy of chimeric (Sotrovimab-framework-Regdanivimab-CDRH3) and other monoclonal antibodies.

| **Lineages** | **Mutant variant** | **Most effective monoclonal antibody** | **Haddock Score** | **Binding affinity ΔG (kcal/mol)** | **Chimeric antibody**  **(Experimentally modified)** | **Haddock Score** | **Binding affinity ΔG (kcal/mol)** | **Lineages** | **Mutant variant** | **Most effective monoclonal antibody** | **Haddock Score** | **Binding affinity ΔG (kcal/mol)** | **Chimeric antibody**  **(Experimentally modified)** | **Haddock Score** | **Binding affinity ΔG (kcal/mol)** |
| --- | --- | --- | --- | --- | --- | --- | --- | --- | --- | --- | --- | --- | --- | --- | --- |
| **B.1.1.7**  **(Alpha lineage)** | A570D | Regdanvimab | -126.1 +/- 20.4 | -13.9 | **Chimeric monoclonal antibody (Grafting Regdanvimab CDRH3 loop inside Sotrovimab)** | **-125.5 +/- 3.6** | **-11.8** | **B.1.617.2**  **(Delta lineage)** | D950N | Sotrovimab | -128.9 +/- 3.6 | -12.7 | **Chimeric monoclonal antibody (Grafting Regdanvimab CDRH3 loop inside Sotrovimab)** | **-143.3 +/- 8.1** | **-14.1** |
|  | D614G | Regdanvimab | -140.3 +/- 3.1 | -10.7 |  | **-141.5 +/- 7.7** | **-16.2** |  | E156G | Regdanvimab | -138.7 +/- 4.1 | -16.1 |  | **-143.3 +/- 6.6** | **-14.8** |
|  | D1118H | Sotrovimab | -132.2 +/- 15.4 | -14.1 |  | **-142.6 +/- 11.2** | **-11.7** |  | F157- | Sotrovimab | -127.2 +/- 10.7 | -11.3 |  | **-103.3 +/- 2.4** | **-12.0** |
|  | H69- | Regdanvimab | -142.3 +/- 12.8 | -12.0 |  | **-104.1 +/- 5.8** | **-12.5** |  | G142D | Sotrovimab | -118.8 +/- 6.9 | -12.0 |  | **-144.7 +/- 2.4** | **-12.5** |
|  | N501Y | Regdanvimab | -121.3 +/- 8.0 | -13.7 |  | **-142.7 +/- 5.4** | **-11.2** |  | L452R | Sotrovimab | -127.6 +/- 8.6 | -11.8 |  | **-113.6 +/- 6.9** | **-12.0** |
|  | P681H | Regdanvimab | -137.2 +/- 8.1 | -10.3 |  | **-102.4 +/- 6.6** | **-12.7** |  | P681R | Regdanvimab | -132.2 +/- 8.2 | -12.3 |  | **-108.6 +/- 2.6** | **-10.7** |
|  | S982A | Regdanvimab | -142.8 +/- 4.1 | -14.7 |  | **-156.2 +/- 5.5** | **-15.7** |  | R158- | Sotrovimab | -132.0 +/- 7.9 | -13.3 |  | **-135.5 +/- 5.5** | **-12.1** |
|  | T716I | Sotrovimab | -122.7 +/- 5.4 | -12.6 |  | **-131.6 +/- 3.3** | **-14.1** |  | T19R | Sotrovimab | -125.5 +/- 1.5 | -13.1 |  | **-127 +/- 3.2** | **-12.5** |
|  | V70- | Regdanvimab | -129.4 +/- 6.0 | -11.2 |  | **-99.1 +/- 5.0** | **-12.0** |  | T478K | Regdanvimab | -130.4 +/- 15.3 | -14.1 |  | **-103.5 +/- 1.6** | **-11.5** |
|  | Y144- | Sotrovimab | -124.3 +/- 6.3 | -10.1 |  | **-140.5 +/- 7.3** | **-15.1** |  | D614G | Regdanvimab | -140.3 +/- 3.1 | -10.7 |  | **-141.5 +/- 7.7** | **-16.2** |

**Table S6:** Analyses of physico-biochemical properties of chimeric (Sotrovimab-framework-Regdanivimab-CDRH3) mAb.

| **Sl. No** | **Features** | **Assessment** |
| --- | --- | --- |
|  | Solubility | 0.503 (Soluble) |
|  | Number of amino acids | 234 |
|  | Molecular weight | 25765.88 Dalton |
|  | Theoretical Isoelectric point (pI) | 9.23 |
|  | Total number of atoms | 3574 |
|  | Formula | C_1141_H_1759_N_313_O_353_S_8_ |
|  | Extinction coefficient | 51590 M^-1^ cm^-1^ |
|  | Estimated half-life | 0.8 hours (mammalian reticulocytes, in vitro) |
|  | Instability index | 32.59 (Stable) |
|  | Aliphatic index | 64.57 |
|  | Grand average of hydropathicity | -0.392 |

**Table S7: Efficacy of chimeric mAb (Sotrovimab-framework-Regdanivimab-CDRH3) against P.1 and B.1.351 lineages.**

| **Sl. No.** | **Lineage of SARS-CoV-2 Strain** | **Interacting Chimeric Monoclonal Antibody** | **Binding Energy (kcal/mol)** | **Haddock 2.4 score^$$^** |
| --- | --- | --- | --- | --- |
|  | P.1 | Sotrovimab-Framework-Regdanivimab-CDRH3 | -13.2 | -50.2 +/- 21.7 |
|  | B.1.351 | Sotrovimab-Framework-Regdanivimab-CDRH3 | -15.0 | -121.5 +/- 14.5 |

^$$^Scores >100 are considered as strong binding/interaction while scores with values <100 are considered as weak binding


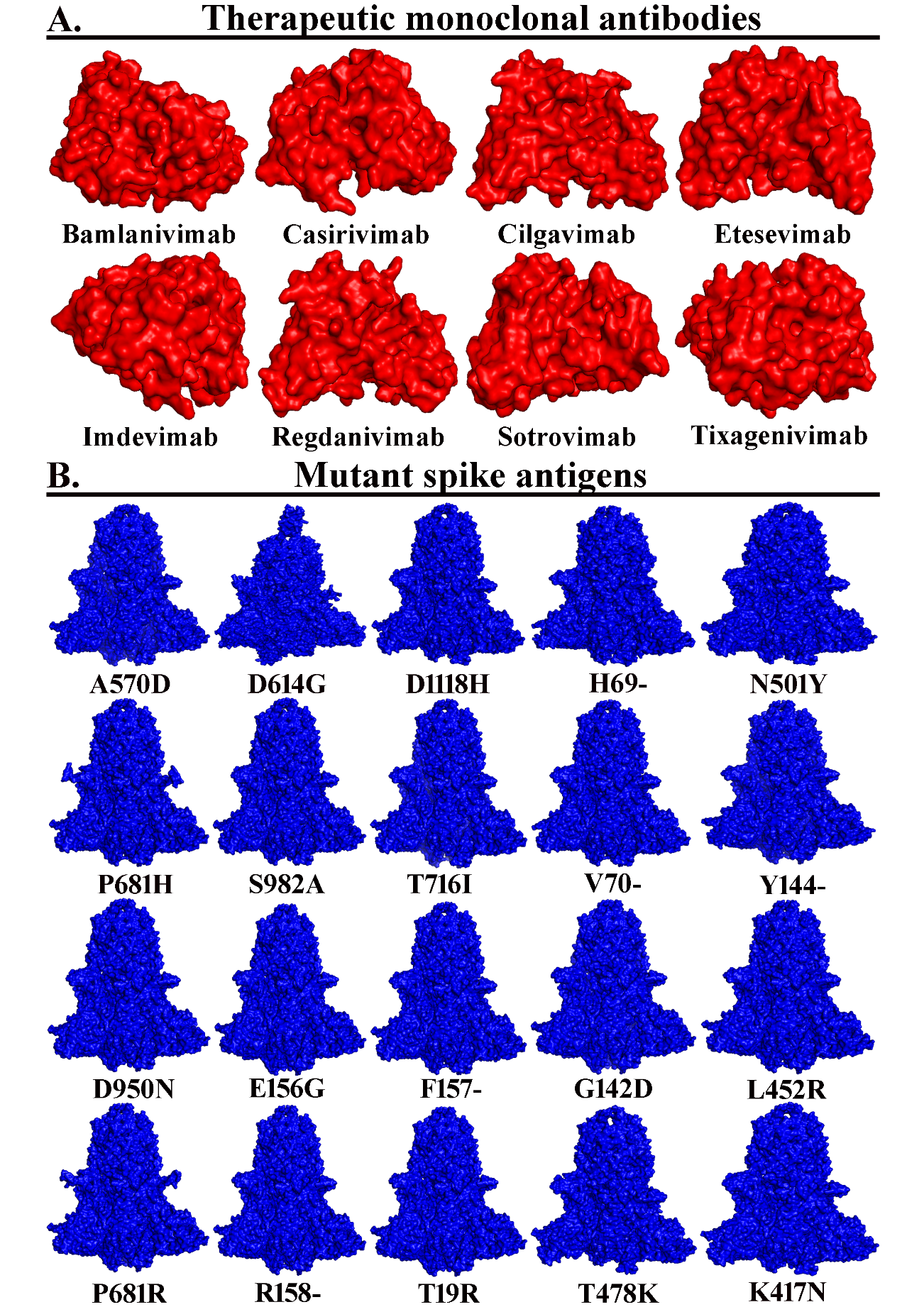


**Figure S1.** Depicting the 3D conformations of A. therapeutic monoclonal antibodies (shown in red) and B. mutant spike antigens (shown in blue) of SARS-CoV-2 Alpha lineage (top two rows) and Delta (bottom two rows).

**
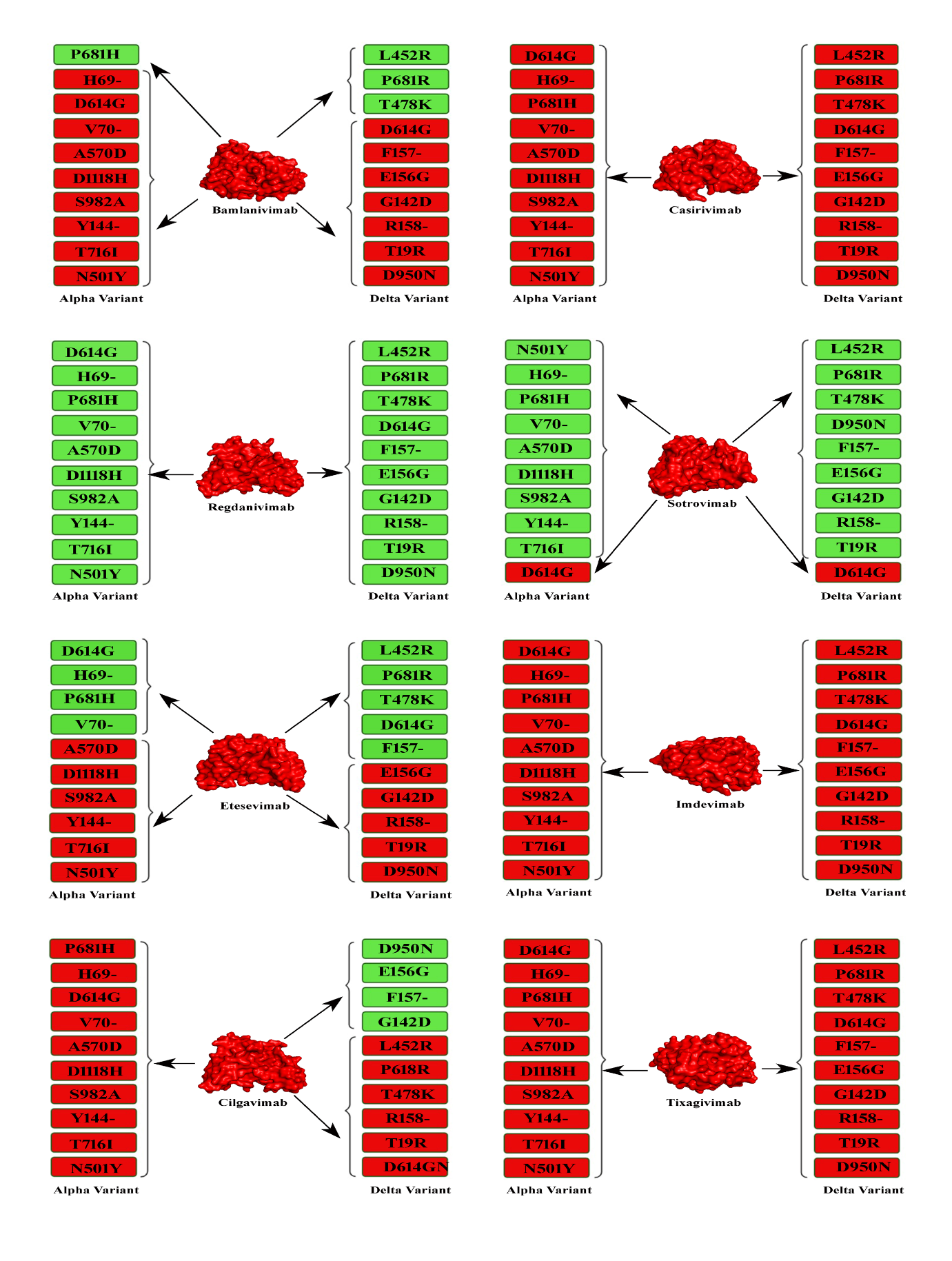
**

**Figure S2. Efficacy of various anti-spike human mAbs in recognizing spike protein variants of SARS-CoV-2 from Alpha and Delta lineages**. Molecular docking-based protein-protein interaction was used for studying the binding efficacy of the mAbs against different spike protein variants. Green colour represents strong binding interactions between the spike protein and the mAbs while weak interactions were represented by the red.

**
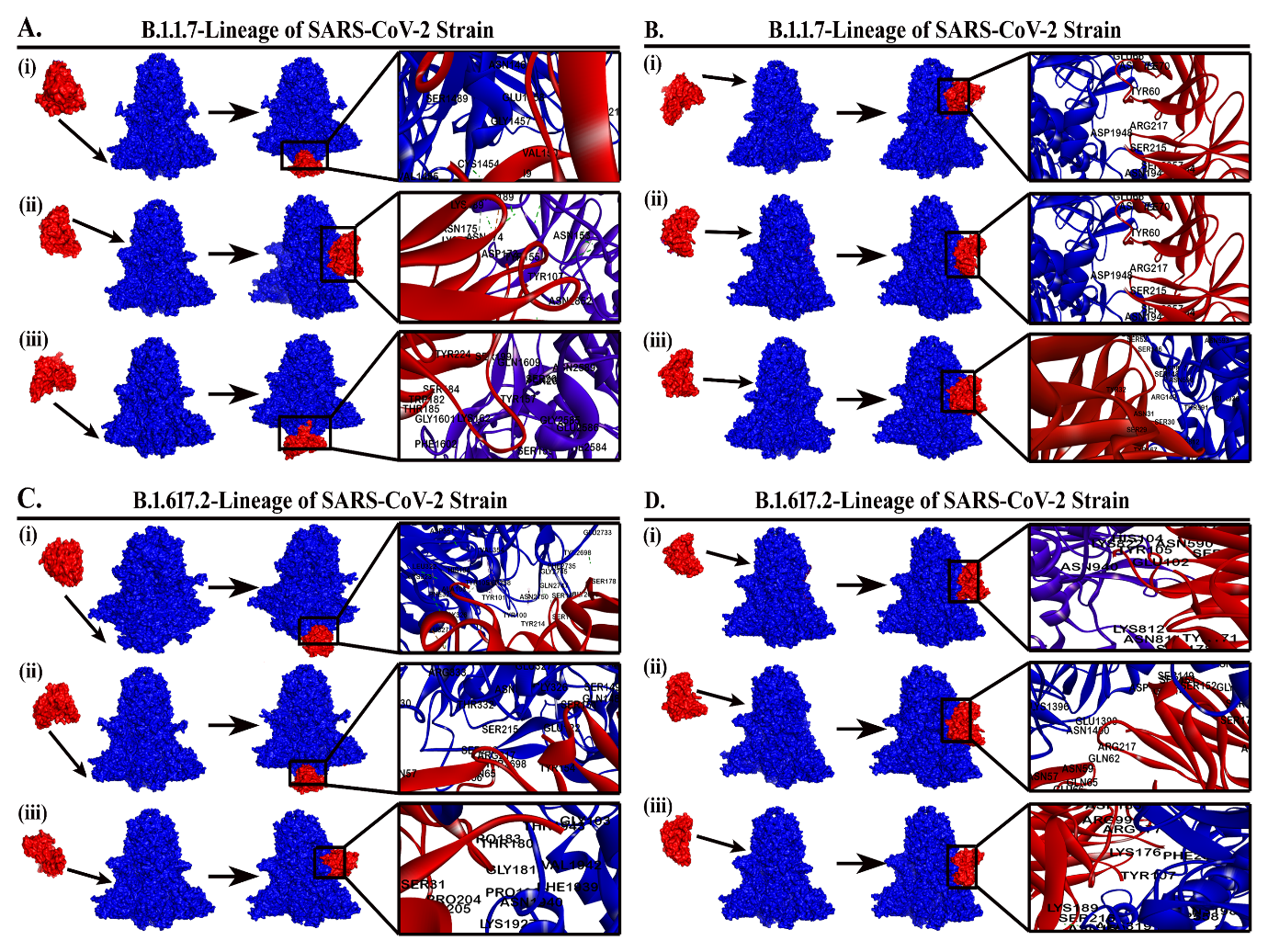
**

**Figure S3. Comparative analyses on the binding pattern and binding topology of different high and low affinity mAbs to SARS-CoV-2 spike protein variants.** A. Interactions between the high affinity mAbs and S protein variants belonging to B.1.1.7 lineage. (i) Interaction between tixagevimab and strain P681H, (ii) regdanvimab and S982A, (ii) cilgavimab and V70-. B. Interactions between the low affinity mAbs and S protein variants belonging to B.1.1.7 lineage. (i) Interaction between cilgavimab and strain T716I, (ii) tixagevimab and S982A, (iii) etesevimab and S982A. C. Interactions between the high affinity mAbs and S protein variants belonging to B.1.617.2 lineage. (i) Interaction between bamlanivimab and strain P681R, (ii) tixagevimab and P681R, (ii) sotrovimab and R158-. D. Interactions between the low affinity mAbs and S protein variants belonging to B.1.617.2 lineage. (i) Interaction between bamlanivimab and strain T19RR, (ii) tixagevimab and G142D, (iii) regdanvimab and T19R.

**
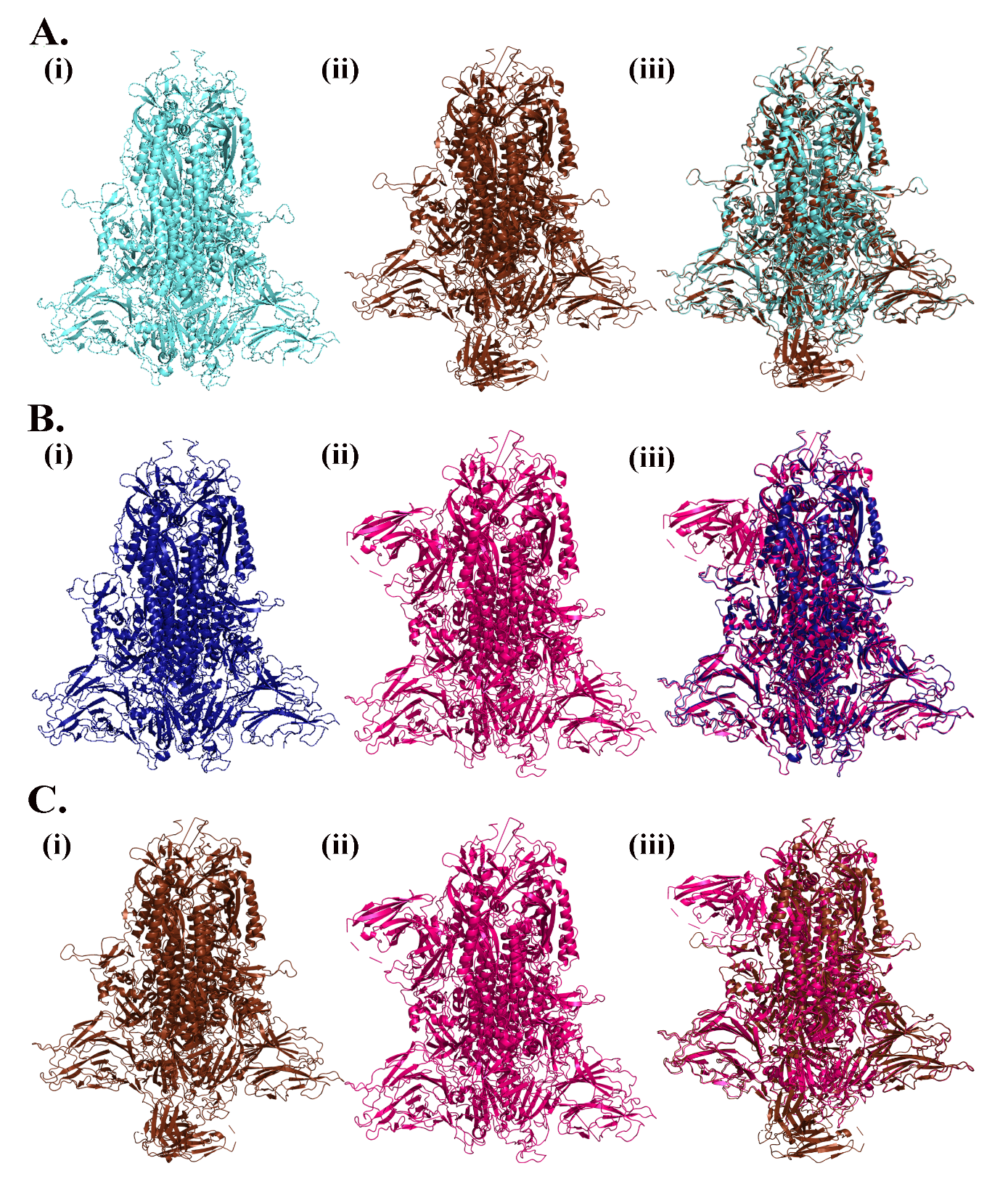
**

**Figure S4. mAb-induced conformational changes in spike glycoprotein.** A. 3D structure of (i) P681H mutant S protein of SARS-CoV-2 (shown in aquamarine) and (ii) tixagevimab-bound P681H (shown in chocolate). (iii) Superimposing tixagevimab-bound P681H (shown in chocolate) and unbound P681H (shown in aquamarine). B. Tertiary structure of (i) T716I mutant S protein of SARS-CoV-2 (denoted as density blue) and (ii) cilgavimab-bound T716I (denoted by hot pink). (iii) Superimposed structure of cilgavimab-bound T716I (denoted as hot pink) and unbound T716I (denoted as density blue). C. 3D conformation of (i) tixagevimab-bound P681H (shown in chocolate) and (ii) cilgavimab-bound T716I complex (shown in hot pink). (iii) Superimposing between tixagevimab-bound P681H (shown in chocolate) and cilgavimab-bound T716I complex (shown in hot pink).

**
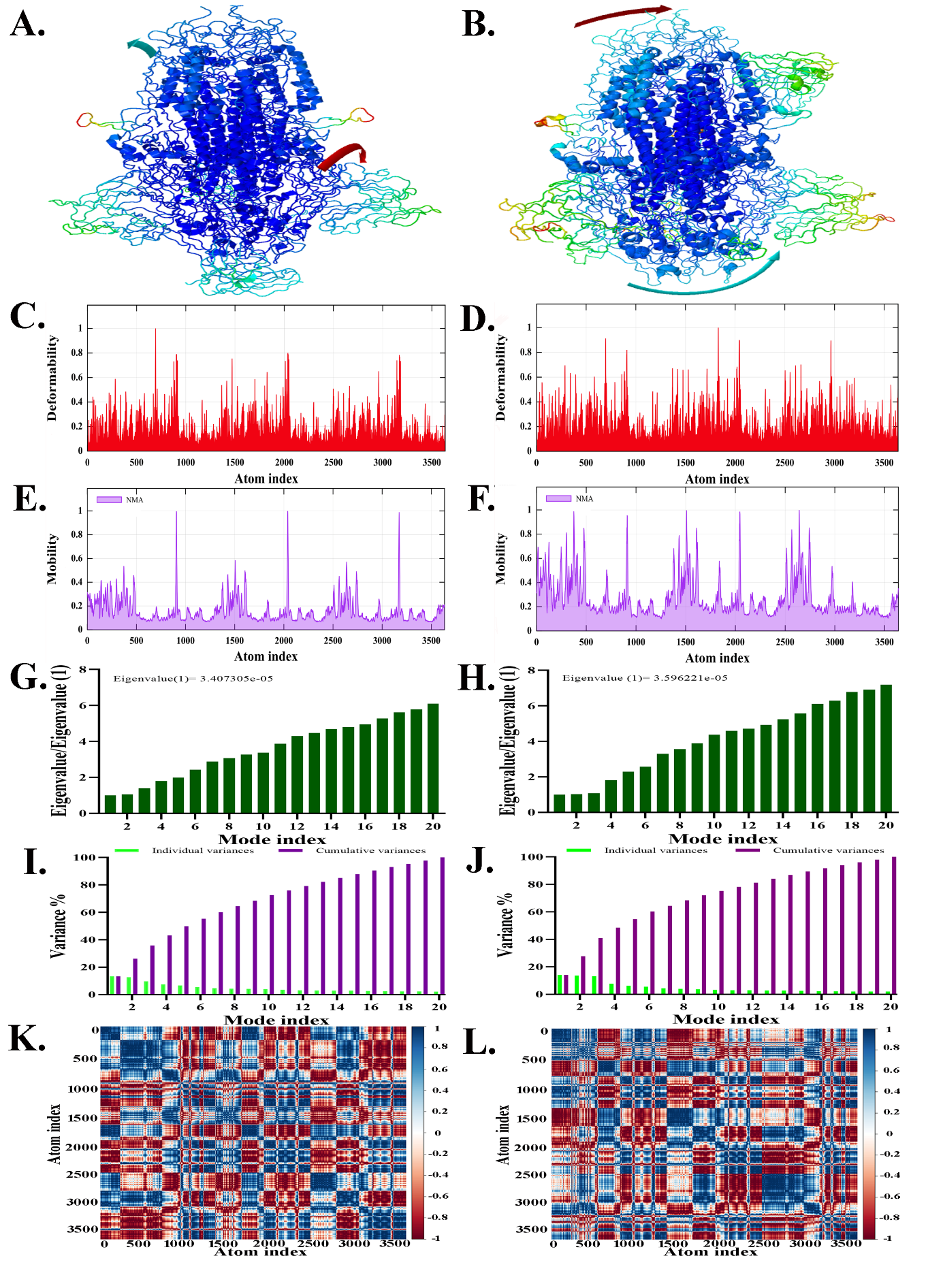
**

**Figure S5. Analyses of the molecular dynamics of strongly- and weekly-bound mAb-spike protein complexes, tixagevimab-bound P681H (left panels) and cilgavimab-bound T716I (right panels) respectively.** A., B. Visualization of molecular motion. C., D. Illustration of deformability level. E., F. Described nature of mobility. G., H. Details of eigenvalues. I., J. Individual and cumulative variance. K., L. Co-variance maps that facilitate understanding of correlated (shown in blue), uncorrelated (shown in white) and anti-correlated (shown in red) motions of Cα atoms.
